# miR‐214‐3p and NCKAP1: A microglial functional dimension of Alzheimer's progression

**DOI:** 10.1002/alz.71903

**Published:** 2026-10-07

**Authors:** Min‐Young Noh, Yeonsil Moon, Sungwoo Kang, Won‐Jin Moon, Hee‐Jin Kim, Sang Won Seo, Sugyeong Ahn, Mi‐Hyeon Kang, Yong Sung Kim, Hyunkee Kim, Jae‐sung Bae, Hee Kyung Jin, Hojin Choi, Seong‐Ho Koh, Kee Hyung Park, Gregory S. Day, Guojun Bu, Seung Hyun Kim

**Affiliations:** ^1^ Department of Neurology, College of Medicine Hanyang University Seoul Republic of Korea; ^2^ Cell Therapy Center Hanyang University Medical Center Seoul Republic of Korea; ^3^ Department of Neurology Konkuk University Medical Center Seoul Republic of Korea; ^4^ Department of Radiology Konkuk University Medical Center Seoul Republic of Korea; ^5^ Department of Neurology Samsung Medical Center, Sungkyunkwan University School of Medicine Seoul Republic of Korea; ^6^ Department of Physiology School of Medicine Kyungpook National University Daegu Republic of Korea; ^7^ Department of Laboratory Animal Medicine College of Veterinary Medicine Kyungpook National University Daegu Republic of Korea; ^8^ Department of Neurology Hanyang University Guri Hospital Hanyang University College of Medicine Guri Republic of Korea; ^9^ Department of Neurology College of Medicine Gachon University Gil Medical Center Incheon Republic of Korea; ^10^ Department of Neurology Mayo Clinic Jacksonville Florida USA; ^11^ Division of Life Science and State Key Laboratory of Nervous System Disorders The Hong Kong University of Science and Technology Clear Water Bay Hong Kong China

**Keywords:** Alzheimer's disease, induced microglia, longitudinal progression, microglial phagocytic exhaustion, miR‐214‐3p, NCK‐associated protein 1 (NCKAP1), plasma biomarkers

## Abstract

**INTRODUCTION:**

Biological Alzheimer's disease (AD) pathology does not fully explain the marked heterogeneity in clinical severity or progression. We investigated whether patient‐derived induced microglia‐like cell (iMG) profiles capture a functional dimension of AD progression beyond conventional plasma biomarkers.

**METHODS:**

We studied 114 participants comprising amyloid‐negative healthy controls (*n =* 18) and amyloid‐positive AD‐continuum groups (cognitively unimpaired, *n =* 2; mild cognitive impairment, *n =* 18; AD dementia, *n =* 76). Plasma biomarkers, patient‐derived iMG phagocytic function, and molecular markers were measured from the same blood draw. Longitudinal clinical follow‐up was available in 102 participants, and paired baseline/follow‐up blood samples were analyzed in a longitudinal biomarker cohort (*n =* 38).

**RESULTS:**

iMG phagocytic capacity progressively declined across the AD continuum, accompanied by reduced NCK‐associated protein 1 (NCKAP1) expression and the strongest association with plasma glial fibrillary acidic protein (GFAP). Baseline NCKAP1 primarily reflected current cognitive impairment, whereas plasma miR‐214‐3p independently predicted faster cognitive decline beyond plasma tau phosphorylated at threonine 217 (p‐tau217), GFAP, neurofilament light chain (NfL), age, and baseline cognition. Within amyloid‐positive AD dementia, plasma miR‐214‐3p identified rapidly progressive disease and substantially improved prognostic discrimination beyond conventional plasma biomarkers. Longitudinally, declining NCKAP1 and increasing plasma miR‐214‐3p tracked the rate of cognitive deterioration, whereas plasma p‐tau217, GFAP, and NfL changed over time but did not track disease tempo.

**DISCUSSION:**

Patient‐derived iMG profiling identifies microglial phagocytic dysfunction as a complementary functional dimension of AD progression beyond conventional plasma biomarkers. Longitudinal microglial dynamics tracked disease tempo more closely than conventional plasma pathology markers and may help interpret biological‐clinical discordance, supporting biologically informed staging, prognosis, and patient stratification in AD.

## Highlights

- Single‐blood‐draw iMG profiling captures microglial dysfunction in AD.
- Microglial phagocytic capacity declines across the AD continuum.
- Reduced NCKAP1 reflects current disease severity in AD.
- Plasma miR‐214‐3p predicts faster progression beyond plasma biomarkers.
- Microglial dynamics track disease tempo beyond plasma biomarkers.

## 1 BACKGROUND

Alzheimer's disease (AD) is increasingly understood not only as a disorder of amyloid and tau accumulation but also as a disease involving immune‐inflammatory dysregulation and impaired microglial function. Microglia, the brain's resident innate immune cells, mediate amyloid beta (Aβ) clearance, synaptic maintenance, and inflammatory regulation,^1^, ^2^ and multiple AD risk genes, including *TREM2*, *BIN1*, *CR1*, and *CD33*, converge on microglial immune pathways.^3^, ^4^ Among these functions, phagocytosis is a key effector process through which microglia remove pathological proteins and damaged synapses to maintain brain homeostasis.^5^, ^6^ Impaired microglial phagocytosis has also been implicated in disease progression across neurodegenerative disorders, including amyotrophic lateral sclerosis (ALS)^7^, ^8^ and Parkinson's disease (PD),^9^, ^10^ suggesting that microglial phagocytosis dysfunction may represent a shared functional mechanism across diseases.^11^

With aging and accumulating AD pathology, microglia may shift from effective clearance toward phagocytic dysfunction, potentially contributing to amyloid accumulation, synaptic injury, neuroinflammation, and clinical progression.^5^, ^12^, ^13^, ^14^, ^15^ However, it remains unclear whether microglial phagocytic dysfunction evolves progressively across the AD continuum, how it relates to established fluid biomarker profiles, and whether it provides patient‐level information relevant to clinical progression.

The 2024 revised criteria for the diagnosis and staging of AD emphasize a biological framework that incorporates inflammatory and immune processes, co‐pathologies, and biological‐clinical staging.^16^ This framework is particularly relevant because biomarker‐defined AD pathology does not always align with clinical severity or clinical trajectory. Such biological‐clinical discordance may reflect cognitive reserve, co‐pathological burden, downstream inflammatory responses, or microglial functional status^17^ and may influence patient selection for disease‐modifying therapies.

Recent amyloid‐targeting trials, including CLARITY AD^18^ and TRAILBLAZER‐ALZ 2,^19^ demonstrated robust amyloid reduction with lecanemab and donanemab, with the greatest clinical benefit observed in earlier‐stage patients. However, clinical responses can differ among patients at similar clinical or biomarker stages, suggesting that amyloid reduction alone may not fully account for clinical outcomes once downstream inflammatory, synaptic, or co‐pathological processes are established. Although biomarkers within an expanded ATN(I) framework may help refine biological staging and patient selection, conventional plasma biomarkers may not fully capture functional microglial states that influence clinical progression.

**RESEARCH IN CONTEXT**

1. **Systematic review**: We searched PubMed and Embase through May 2025 for studies examining microglial phagocytosis, plasma biomarkers, and induced microglia in AD. Previous studies relied primarily on *post mortem* tissue, animal models, or isolated biomarker measurements. To our knowledge, no study has integrated plasma biomarkers with patient‐derived iMG function from the same blood draw to investigate biological‐clinical discordance across the AD continuum.
2. **Interpretation**: The 2024 revised AD framework recognizes that biomarker‐defined pathology does not fully explain clinical severity or disease progression. In this study, same‐patient iMG profiling identified progressive microglial phagocytic dysfunction across the AD continuum. Baseline NCKAP1 primarily reflected current disease severity, whereas plasma miR‐214‐3p predicted subsequent cognitive decline beyond conventional plasma biomarkers. Longitudinally, changes in microglial markers – but not plasma pathology markers – tracked disease tempo, supporting microglial dysfunction as a complementary functional dimension of AD progression and helping operationalize the neuroinflammatory “I” component at the cellular level.
3. **Future directions**: Future studies should validate these findings in independent cohorts, determine their longitudinal stability, and evaluate whether microglial dysfunction profiling can improve biological‐clinical stratification and therapeutic targeting in AD clinical trials.

Plasma phosphorylated tau at threonine 217 (p‐tau217) has emerged as the most accurate plasma biomarker of AD pathology,^20^, ^21^ while glial fibrillary acidic protein (GFAP) and neurofilament light (NfL) provide complementary information on astrocytic reactivity and neuronal injury, respectively.^22^, ^23^, ^24^, ^25^ Yet whether microglial phagocytic dysfunction is reflected in these biomarker patterns, modified by apolipoprotein E ε4 allele (APOE ε4), or associated with clinical progression remains incompletely understood. This question is particularly relevant because APOE ε4, the strongest genetic risk factor for AD,^26^ has been linked to altered microglial lipid handling and phagocytic‐lysosomal function.^27^, ^28^

To address this gap, we generated peripheral blood mononuclear cell (PBMC)‐derived induced microglia‐like cells (iMGs)^29^ and profiled microglial phagocytic function across healthy controls (HCs) and the amyloid‐positive AD continuum. We then examined whether iMG phagocytic dysfunction profiles were associated with clinical stage, amyloid status, APOE ε4 status, established plasma and CSF biomarkers, and longitudinal clinical progression. Our previous studies using the same patient‐derived iMG platform in ALS identified NCK‐associated protein 1 (NCKAP1), a regulator of actin remodeling and phagocytic machinery,^7^, ^30^ as a molecular determinant of impaired microglial phagocytosis associated with rapid disease progression, with the restoration of NCKAP1 rescuing the phagocytic defect.^7^ We subsequently identified plasma miR‐214‐3p as a NCKAP1‐targeting microRNA associated with ALS disease progression.^8^ Based on these findings, we hypothesized that the NCKAP1‐associated phagocytic axis might represent a broader functional axis of microglial dysfunction beyond ALS^11^ and, therefore, examined NCKAP1 and plasma miR‐214‐3p across the AD continuum.

Overall, this study tested whether patient‐derived microglial phagocytic dysfunction profiles provided adjunctive functional markers for interpreting biological‐clinical discordance and refining patient stratification in AD.

## 2 METHODS

### 2.1 Study design and participants

This study was designed around same‐patient, same‐draw profiling, in which plasma biomarkers and patient‐specific microglial functions were assessed in parallel from a single venous blood sample obtained from each participant (Figure 1A). This design enabled direct within‐individual evaluation of the relationship between circulating biomarker profiles and iMG function, while minimizing temporal and inter‐individual confounding that may arise when plasma biomarkers and cellular phenotypes are assessed in separate cohorts or at different time points.

**FIGURE 1:**
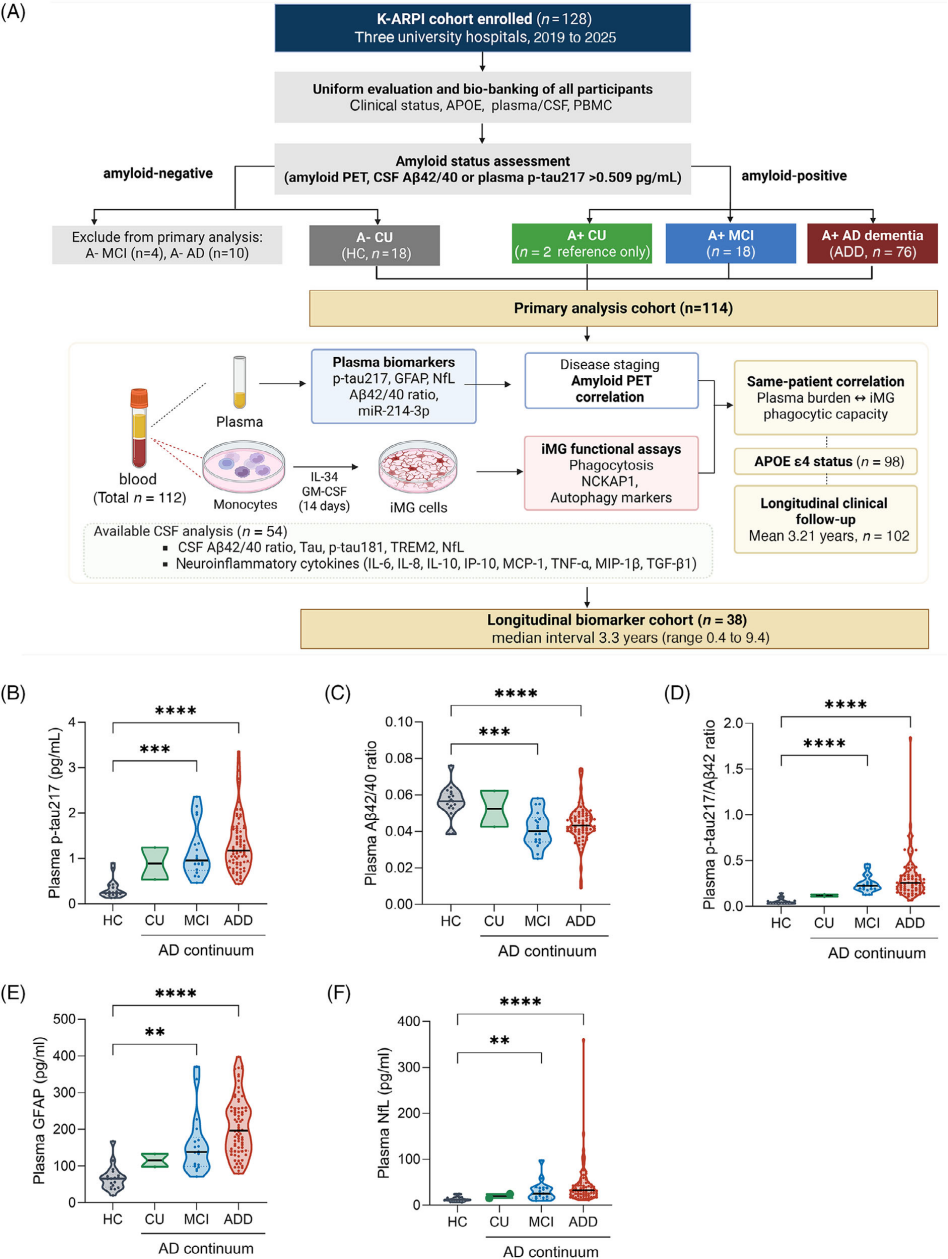
Same‐patient profiling integrates plasma biomarkers and patient‐derived microglial analyzes across the AD continuum. (A) Study design and participant flow. Amyloid status was determined by amyloid PET or CSF Aβ42/40 ratio, with plasma p‐tau217 used as a supportive indicator only when neither was available. The primary cohort comprised 114 participants: amyloid‐negative cognitively unimpaired controls (HC, *n =* 18) and the amyloid‐positive AD continuum (amyloid‐positive CU, *n =* 2; MCI, *n =* 18; ADD, *n =* 76). A single blood sample from each participant was processed in parallel for plasma biomarker measurement and generation of induced microglia‐like cells (iMGs). Additional analyses included CSF biomarker (*n =* 54), APOE ε4 stratification (*n =* 98), longitudinal clinical follow‐up (*n =* 102), and repeat blood sampling for longitudinal biomarker analyzes (*n =* 38). Created with BioRender. (B) Plasma p‐tau217, (C) Aβ42/40 ratio, (D) p‐tau217/Aβ42 ratio, (E) GFAP, and (F) NfL across the AD continuum. Violin plots show individual participants (gray = HC, green* =* amyloid‐positive CU, blue = MCI, red = ADD); horizontal lines indicate the median and interquartile range (IQR). Group comparisons were performed using the Kruskal–Wallis test with Dunn–Bonferroni post hoc correction. The amyloid‐positive CU group (*n =* 2) is shown for descriptive purposes only and was excluded from statistical comparisons. Statistical significance: *p < 0.05, **p < 0.01, ***p < 0.001, ****p < 0.0001. ADD, amyloid‐positive Alzheimer's disease dementia; APOE ε4, apolipoprotein E ε4; AUC, area under the curve; Aβ42, amyloid beta 42; Aβ42/40, amyloid beta 42/40 ratio; CDR, Clinical Dementia Rating; CDR‐SB, CDR Sum of Boxes; CI, confidence interval; CSF, cerebrospinal fluid; CU, amyloid‐positive cognitively unimpaired; GFAP, glial fibrillary acidic protein; GM‐CSF, granulocyte‐macrophage colony‐stimulating factor; HC, healthy control (amyloid‐negative cognitively unimpaired); iMG, induced microglia‐like cell; IQR, interquartile range; MCI, amyloid‐positive mild cognitive impairment; MMSE, Mini‐Mental State Examination; NfL, neurofilament light chain; p‐tau217, phosphorylated tau 217; PET, positron emission tomography; RFU, relative fluorescence units; RQ, relative quantification.

A total of 128 participants, including 20 cognitively unimpaired (CU) individuals and 108 individuals with cognitive complaints, were prospectively recruited from Hanyang University Medical Center, Konkuk University Medical Center, and Samsung Medical Center between 2017 and 2025 through the Korean AD Research Platform Initiative based on the Immune‐inflammatory Biomarkers (K‐ARPI) platform,^31^ a South Korean precision‐medicine network, according to National Institute on Aging‐Alzheimer's Association (NIA‐AA) criteria.^16^, ^32^ All participants underwent standardized clinical evaluation and biobanking, with each venous blood sample processed in parallel into plasma and PBMCs, together with cerebrospinal fluid (CSF) when available.

Amyloid positivity was determined using amyloid PET or CSF Aβ42/40 ratio when available; plasma p‐tau217 (>0.509 pg/mL) was used only when neither PET nor CSF data were available. The primary analysis cohort comprised 114 participants, including amyloid‐negative CU (HC, *n =* 18) and individuals across the amyloid‐positive AD continuum – amyloid‐positive CU (*n =* 2; reference only), amyloid‐positive mild cognitive impairment (MCI, *n =* 18), and amyloid‐positive AD dementia (ADD, *n =* 76) (Table 1).

**TABLE 1:** Baseline participant characteristics and integrated plasma–iMG measurements.

Characteristic	Amyloid‐negative CU (HC, *n =* 18)	Amyloid‐positive CU (*n =* 2)	Amyloid‐positive MCI (*n =* 18)	Amyloid‐positive AD dementia (ADD, *n =* 76)	Overall *p*
**Demographics**					
Age, years^†^	64.4 ± 12.4^c^	78, 69	72.9 ± 9.3	73.4 ± 8.4^a^	0.017
Sex, male	33.3%	2/2	38.9%	47.4%	0.504
Education, years^†^	9.2 ± 4.0	8.0, 6.0	9.8 ± 5.7	9.1 ± 4.8	0.905
APOE ε4 carrier	1/12 (8.3%)^b^	1/1^#^	12/16 (75.0%)^a^	32/69 (46.4%)	0.002
**Clinical severity**					
MMSE	29.0 [28.0, 30.0]^c^	30, 29	25.5 [24.0, 27.0]^c^	17.0 [12.8, 20.0]^a^, ^b^	<0.001
CDR global score	0.0 [0.0, 0.0]^c^	0.0, 0.0	0.5 [0.5, 0.5]^c^	1.0 [1.0, 2.0]^a^ ^,^ ^b^	<0.001
CDR‐SB	0.0 [0.0, 0.4]^c^	0.0, 0.0	1.0 [0.5, 1.9]^c^	6.0 [4.5, 10.0]^a^ ^,^ ^b^	<0.001
Amyloid positive^§^	0/18 (0.0%)	2/2	18/18 (100%)	76/76 (100%)	—
**Plasma biomarkers (same blood draw as iMGs)**					
p‐tau217, pg/mL	0.240 [0.200, 0.396]^b^ ^,^ ^c^	1.241, 0.534	0.954 [0.783, 1.324]^a^	1.174 [0.906, 1.651]^a^	<0.001
GFAP, pg/mL	65.7 [43.5, 73.7]^b^ ^,^ ^c^	133.0, 98.0	137.8 [100.6, 169.4]^a^	196.2 [141.7, 257.1]^a^	<0.001
NfL, pg/mL	11.69 [9.36, 13.89]^b^ ^,^ ^c^	24.1, 15.5	25.30 [16.83, 37.62]^a^	32.83 [24.08, 48.64]^a^	<0.001
Aβ42, pg/mL	6.19 [5.08, 6.54]^b^ ^,^ ^c^	9.62, 4.94	4.31 [3.23, 5.47]^a^	4.58 [3.80, 5.76]^a^	0.004
Aβ42/40 ratio	0.057 [0.053, 0.060]^b^ ^,^ ^c^	0.062, 0.043	0.040 [0.035, 0.047]^a^	0.043 [0.040, 0.049]^a^	<0.001
p‐tau217/Aβ42 ratio	0.042 [0.031, 0.059]^b^ ^,^ ^c^	0.129, 0.108	0.225 [0.191, 0.266]^a^	0.255 [0.182, 0.344]^a^	<0.001
**iMG functional assays (same blood draw as plasma)**					
Aβ42 phago (RFU/cell)	1.998 [1.691, 2.213]^c^	1.620, 1.780	1.495 [1.211, 2.330]^c^	0.970 [0.752, 1.285]^a^ ^,^ ^b^	<0.001
Bead phago (beads/cell)	87.2 [77.9, 102.9]^c^	97.3, 77.1	70.9 [63.8, 83.0]^c^	50.1 [35.5, 63.6]^a^ ^,^ ^b^	<0.001
*E. coli* phago (RFU/cell)	88.0 [83.2, 90.9]^c^	84.3, 80.0	68.2 [66.0, 82.5]^c^	55.5 [40.0, 65.9]^a^ ^,^ ^b^	<0.001
NCKAP1 (RQ)	1.35 [1.08, 1.61]^c^	1.28, 1.08	0.96 [0.78, 1.19]	0.66 [0.40, 0.97]^a^	<0.001
Rubicon (RQ)	0.367 [0.228, 0.610]^b^ ^,^ ^c^	0.348, 0.334	5.324 [0.841, 12.613]^a^ ^,^ ^c^	0.886 [0.370, 4.086]^a^ ^,^ ^b^	<0.001
**CSF markers (subset, *n =* 54)** ^‡^					
CSF Aβ42/40 ratio	0.156 [0.129, 0.180] (*n =* 4)^b^ ^,^ ^c^	—	0.090 [0.079, 0.098] (*n =* 5)^a^	0.089 [0.073, 0.101] (*n =* 45)^a^	0.006
CSF p‐tau181, pg/mL	40.2 [33.7, 50.8] (*n =* 4)^c^	—	98.7 [57.4, 99.8] (*n =* 5)	135.1 [86.1, 160.9] (*n =* 45)^a^	0.007
CSF sTREM2, pg/mL	1477 [1128, 1696] (*n =* 4)^c^	—	2654 [2466, 2718] (*n =* 5)	2571 [2044, 2944] (*n =* 45)^a^	0.014
CSF TGF‐β1, pg/mL	1047 [1007, 1127] (*n =* 4)^b^ ^,^ ^c^	—	652 [555, 742] (*n =* 5)^a^	672 [545, 804] (*n =* 45)^a^	0.008

Note: Data are median [IQR] for continuous variables or *n*/*N* (%) for categorical variables, unless otherwise indicated. Primary comparisons were performed among amyloid‐negative cognitively unimpaired controls (HC), amyloid‐positive MCI, and amyloid‐positive AD dementia (ADD). The amyloid‐positive CU group (*n =* 2) is shown for descriptive reference only and was not included in statistical testing. Overall *p*: Kruskal–Wallis test (continuous) or chi‐squared test (categorical). Superscript letters denote statistically significant pairwise post hoc differences (Dunn's test with Bonferroni correction for continuous variables; Fisher's exact test with Bonferroni correction for categorical variables; *p* < 0.05). Of note, plasma p‐tau217, Aβ42/40, and p‐tau217/Aβ42 carry no ^c^/^b^ marker between MCI and ADD (no significant MCI–ADD difference; plateau), whereas iMG Aβ42, bead, and *E. coli* phagocytosis are marked ^a^^,^^b^ in ADD, indicating continued microglial staging beyond the plasma plateau.

^a^ Significantly different from HC.

^b^ From A+MCI.

^┼^ From A+ADD. Biomarker group comparisons are also depicted graphically in Figures 1, 2, 3 and Figure S2.

^†^ Age and education are mean ± SD.

^‡^ CSF available in a subset (*n =* 54, predominantly ADD); comparisons involving HC (*n =* 4) and MCI (*n =* 5) are exploratory (Figure S2).

^§^ Amyloid positivity by amyloid PET (BAPL 2‐3) or CSF, with plasma p‐tau217 (> 0.509 pg/mL) as a supportive indicator only when neither was available. Amyloid‐positive CU (*n =* 2) reference only; amyloid‐negative MCI (*n* = 4) and ADD (*n* = 10) were excluded from the primary analysis (sensitivity analyses, Figure S3).

^#^ One amyloid‐positive CU participant had missing APOE genotype.

Abbreviations: ADD, amyloid‐positive Alzheimer's disease dementia; APOE ε4, apolipoprotein E ε4 allele; Aβ42, amyloid beta 42; Aβ42/40, amyloid beta 42/40 ratio; BAPL, brain amyloid plaque load; CDR, Clinical Dementia Rating; CDR‐SB, CDR Sum of Boxes; CSF, cerebrospinal fluid; CU, amyloid‐positive cognitively unimpaired; GFAP, glial fibrillary acidic protein; HC, healthy control (amyloid‐negative cognitively unimpaired); iMGs, induced microglia‐like cells; IQR, interquartile range; MCI, amyloid‐positive mild cognitive impairment; MMSE, Mini‐Mental State Examination; NCKAP1, non‐catalytic region of tyrosine kinase‐associated protein 1; NfL, neurofilament light chain; PET, positron emission tomography; p‐tau217, phosphorylated tau 217; RFU, relative fluorescence units; RQ, relative quantification; Rubicon, RUN domain Beclin‐1‐interacting and cysteine‐rich‐containing protein; sTREM2, soluble triggering receptor expressed on myeloid cells 2.

Clinical status was assessed using the Mini‐Mental State Examination (MMSE), global Clinical Dementia Rating (CDR), and CDR Sum of Boxes (CDR‐SB). Among the 114 participants, 102 had longitudinal follow‐up data with at least two MMSE and CDR‐SB assessments. Longitudinal clinical progression was evaluated using annualized changes in MMSE and CDR‐SB scores (ΔMMSE/year and ΔCDR‐SB/year; mean follow‐up 3.21 years, SD 1.64; range 0.4 to 9.4). Rapid clinical decline was defined a priori as an annualized MMSE decline ≥4 points and CDR‐SB increase ≥2.5 points based on published criteria for clinically meaningful progression.^33^, ^34^, ^35^

In a subset of participants, repeat blood samples were obtained during follow‐up for longitudinal assessment of plasma biomarkers and patient‐specific iMG measures. To minimize batch effects, baseline PBMCs were cryopreserved and processed in parallel with the corresponding follow‐up samples in the same experimental batch, including re‐derivation of iMGs. The longitudinal biomarker cohort included 38 participants with paired baseline and follow‐up samples (median interval, 3.3 years [range, 0.4 to 9.4]: three HC, four amyloid‐positive MCI, and 31 amyloid‐positive ADD).

APOE ε4 genotyping was available in 98 of the participants (ε4− *n =* 53, ε4+ *n =* 45), and CSF samples were available in a subset (*n =* 54). This study was conducted in accordance with the World Medical Association's Declaration of Helsinki and approved by the Institutional Review Boards (IRBs) of Hanyang University Hospital (HYUH IRB 2017‐01‐043, 2018‐08‐020, 2021‐02‐034), Konkuk University Medical Center (KUH1170180), and Samsung Medical Center (2018‐07‐016); all participants provided written informed consent.

### 2.2 Amyloid PET imaging and definition of amyloid positivity

Amyloid PET was performed using ^18^F‐florbetaben at three participating centers following standard acquisition protocols. Amyloid positivity was determined using the Brain Amyloid‐β Plaque Load (BAPL) visual rating system, with scores of 2 or 3 considered positive.^36^ Images were independently reviewed by two board‐certified nuclear medicine physicians blinded to clinical information, and consensus ratings were used for analyses. For cohort classification, amyloid positivity was defined by amyloid PET, CSF Aβ42/Aβ40 ratio, or plasma p‐tau217 when PET or CSF data were unavailable. Additional details are provided in the Supplementary Methods and Figure S1.

### 2.3 Apolipoprotein E (APOE) genotyping

Genomic DNA was isolated from peripheral blood, and APOE genotyping was performed using a validated real‐time polymerase chain reaction (PCR)‐based assay. APOE ε4 carrier status was defined by the presence of at least one ε4 allele and was analyzed as a binary variable (carrier vs non‐carrier).

### 2.4 Generation of human iMGs from PBMCs

Human iMGs were generated from PBMCs as previously described.^29^ This approach generates cells that recapitulate key transcriptomic and functional features of human microglia.^7^, ^37^, ^38^ Briefly, adherent monocytes isolated from PBMCs were differentiated into iMGs by culture with GM‐CSF and IL‐34 for 14 days. Detailed culture procedures are provided in the Supplementary Methods.

### 2.5 iMG phagocytic capacity and molecular characterizations

iMG phagocytic capacity was assessed using three complementary substrates: fluorescent latex beads (bulk phagocytosis), fluorescently labeled Aβ42 fibrils (disease‐relevant clearance),^39^ and pHrodo‐conjugated *Escherichia coli* (*E. coli *) bioparticles (innate immune phagocytosis). Following incubation, phagocytic uptake was quantified by fluorescence microscopy.

Gene expression analyses were performed by quantitative real‐time PCR as previously described.^40^ NCKAP1 expression was quantified as a marker of phagocytic competence. In addition, expressions of autophagy‐ and lysosome‐related genes, including RUBCN, EIF5A, TFEB, TFE3, LAMP1, and COX2, were assessed to characterize pathways associated with phagocytic dysfunction. Gene expression levels were normalized to GAPDH and expressed as relative quantification values.^41^, ^42^ Detailed phagocytosis and gene expression procedures are described in the Supplementary Methods. Assay information is summarized in Table S1.

### 2.6 Plasma biomarker measurements

Plasma samples were obtained by morning venipuncture under fasting conditions and processed according to the Alzheimer's Disease Neuroimaging Initiative 2 procedures.^43^ Plasma samples were collected under fasting conditions and stored at −80°C until analysis. Plasma p‐tau217 was measured using the Single‐Molecule Array (Simoa) Human ALZpath p‐tau217 Advantage PLUS assay. Plasma GFAP, NfL, Aβ40, and Aβ42 were measured using the Simoa Neurology 4‐Plex E Advantage PLUS assay. All samples were analyzed on the Simoa HD‐X platform and measured in duplicate with coefficients of variation below 10%.

Plasma miR‐214‐3p was quantified by quantitative real‐time PCR following small‐RNA extraction (miRNeasy Serum/Plasma Advanced Kit, Qiagen) and reverse transcription (miRCURY LNA RT Kit, Qiagen), including spike‐in cel‐miR‐39 as an external control. qPCR was performed using miRCURY LNA SYBR Green chemistry (Qiagen). Relative expression levels were calculated using the ΔΔCt method with miR‐103‐3p and miR‐423‐5p as endogenous reference controls and expressed as relative quantification (RQ) values.^8^ Detailed reagent information, assay identifiers, and primer sequences are provided in Table S1.

### 2.7 CSF biomarker measurements

CSF samples were collected by lumbar puncture in polypropylene tubes and stored at −80°C until analysis. In participants with available CSF samples (*n =* 54), soluble TREM2 was measured using a Human TREM2 SimpleStep ELISA kit (Abcam).^44^, ^45^ A panel of neuroinflammatory cytokines (IL‐6, IL‐8, IL‐10, IP‐10, MCP‐1, TNF‐α, MIP‐1β, and TGF‐β1) was quantified using multiplex immunoassays (Millipore). CSF Aβ42/40 ratio, total tau, and p‐tau181 were measured using the MILLIPLEX Human Amyloid Beta and Tau Panel,^46^ and CSF NfL concentrations were determined using a commercial ELISA assay.^47^ Detailed assay information is summarized in Table S1.

### 2.8 Statistical analysis

Continuous variables are presented as median (interquartile range [IQR]) and categorical variables as *n* (%). Group comparisons were performed using the Kruskal‐Wallis test with Dunn‐Bonferroni post hoc correction, Mann‐Whitney U test, chi‐squared test, or Fisher's exact test, as appropriate. Associations between variables were assessed using Spearman rank correlation. Partial correlation and regression analyses were performed to evaluate associations between iMG measures and longitudinal clinical progression after adjustment for relevant covariates. APOE ε4‐stratified and interaction analyses were conducted to assess potential effect modification by genotype. Receiver operating characteristic (ROC) analyses were used to evaluate discrimination of amyloid PET positivity and rapidly progressive AD (rpAD).

For the longitudinal biomarker cohort, baseline and follow‐up measurements were compared using the Wilcoxon signed‐rank test, and associations between annualized biomarker and clinical changes were assessed using the Spearman correlation.

All tests were two‐sided, and *p *< 0.05 was considered statistically significant. Except for Dunn‐Bonferroni correction applied to post hoc group comparisons, *p* values were not adjusted for multiple testing and should be interpreted as exploratory. Statistical analyses were performed using GraphPad Prism version 10.0 and R version 4.3.0. Additional details are provided in the Supplementary Methods.

## 3 RESULTS

### 3.1 Participant characteristics and same‐patient plasma biomarker staging

Following amyloid classification, 114 participants were included in the primary analysis, comprising HCs (*n =* 18) and individuals across the amyloid‐positive AD continuum (A+ CU, *n =* 2; A+ MCI, *n =* 18; A+ ADD, *n* = 76) (Table 1). Demographic and clinical characteristics are summarized in Table 1. APOE ε4 carrier frequency increased across the AD continuum (*p *= 0.002), whereas MMSE, CDR, and CDR‐SB scores reflected progressively greater cognitive impairment and disease severity (all *p *< 0.001).

Consistent with the study design (Figure 1A), plasma biomarkers and patient‐derived iMG phenotypes were evaluated in parallel from the same blood sample obtained from each participant.

Plasma p‐tau217, p‐tau217/Aβ42 ratio, GFAP, and NfL increased progressively across the AD continuum, whereas plasma Aβ42/40 ratio decreased (all *p *< 0.0001; Figure 1B–F). Similar findings were observed in the amyloid PET subset, in which plasma biomarkers discriminated PET positivity (Figure S1 in supporting information). These results confirmed that plasma biomarker profiles tracked with increasing AD pathological burden across the cohort.

### 3.2 Progressive microglial phagocytic exhaustion across AD continuum

To assess patient‐specific microglial functional capacity, phagocytosis was measured in iMGs using three complementary substrates: Aβ42 fibrils, fluorescent beads, and *E. coli* bioparticles. Across all three substrates, phagocytic capacity declined progressively across the AD continuum (all Kruskal‐Wallis *p *< 0.0001; Figure 2A–C). The impairment was most pronounced at the dementia stage, with significantly lower phagocytic activity in ADD compared with HC and MCI. Similar findings were observed when amyloid‐negative cognitively impaired participants were included in the analysis (all *p *< 0.0001; Figure S2), indicating that the observed decline was not driven solely by the primary cohort definition.

**FIGURE 2:**
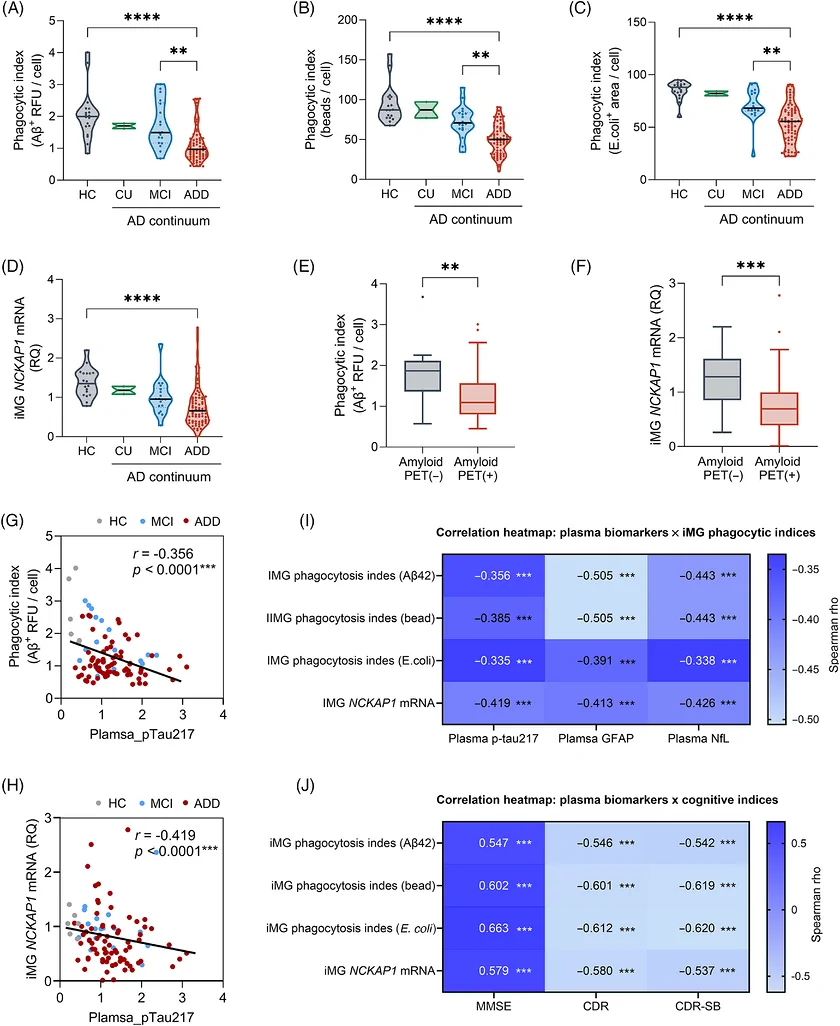
Progressive microglial phagocytic dysfunction across the AD continuum is accompanied by declining NCKAP1 expression. (A) iMG Aβ42 fibril phagocytic index, (B) bead phagocytic index, and (C) *E. coli* phagocytic index across disease stages (gray = HC, green* =* amyloid‐positive CU, blue = MCI, red = ADD). (D) iMG NCKAP1 mRNA expression across disease stages. (E) Aβ42 phagocytic index and (F) NCKAP1 mRNA expression in amyloid PET‐negative (*n =* 15) and PET‐positive (*n =* 60) participants. (G) Association between plasma p‐tau217 and iMG Aβ42 phagocytic capacity. (H) Association between plasma p‐tau217 and iMG NCKAP1 expression. (I) Correlation heatmap showing associations between iMG functional indices and plasma biomarkers (p‐tau217, GFAP, and NfL). (J) Correlation heatmap showing associations between iMG functional indices and clinical measures (MMSE, CDR, and CDR‐SB). For scatter plots, each point represents one participant and lines indicate linear regression fits. Statistical significance: *p < 0.05, **p < 0.01, ***p < 0.001, ****p < 0.0001. ADD, amyloid‐positive Alzheimer's disease dementia; Aβ42, amyloid beta 42; CDR, Clinical Dementia Rating; CDR‐SB, CDR Sum of Boxes; CU, amyloid‐positive cognitively unimpaired; GFAP, glial fibrillary acidic protein; HC, healthy control (amyloid‐negative cognitively unimpaired); iMG, induced microglia‐like cell; IQR, interquartile range; MCI, amyloid‐positive mild cognitive impairment; MMSE, Mini‐Mental State Examination; NCKAP1, non‐catalytic region of tyrosine kinase‐associated protein 1; NfL, neurofilament light chain; p‐tau217, phosphorylated tau 217; PET, positron emission tomography; RFU, relative fluorescence units; RQ, relative quantification.

NCKAP1 mRNA expression also decreased progressively across the AD continuum (*p *< 0.0001; Figure 2D). Given the established role of NCKAP1 in actin remodeling and phagocytic cup formation, its reduction was mechanistically consistent with the impaired phagocytic function observed across all three substrates.^7^, ^48^ Among participants with amyloid PET imaging, PET‐positive individuals exhibited lower Aβ42 phagocytosis and reduced NCKAP1 expression compared with PET‐negative individuals (Figure 2E,F).

Same‐patient correlation analyses demonstrated inverse relationships between plasma biomarker burden and iMG function. Plasma p‐tau217 was negatively correlated with both Aβ42 phagocytosis and NCKAP1 expression (Figure 2G,H). Across the biomarker‐iMG correlation matrix, plasma GFAP showed the strongest inverse associations with iMG functional measures, including Aβ42 phagocytosis, bead phagocytosis, and NCKAP1 expression (Figure 2I). Plasma NfL and p‐tau217 also showed significant inverse correlations with multiple phagocytic indices.

In parallel, iMG phagocytic function and NCKAP1 expression were positively associated with cognitive performance, including MMSE, CDR, and CDR‐SB scores (Figure 2J). Collectively, these findings identify a coordinated relationship among increasing plasma biomarker burden, reduced NCKAP1 expression, impaired microglial phagocytic capacity, and worsening clinical severity across the AD continuum.

### 3.3 Plasma miR‐214‐3p as a blood‐based correlate of NCKAP1‐linked microglial phagocytic exhaustion

Plasma miR‐214‐3p, a predicted NCKAP1‐targeting microRNA,^8^ was evaluated as a blood‐based correlate of microglial phagocytic dysfunction.^11^ Plasma miR‐214‐3p levels were highest in ADD, significantly exceeding HC, amyloid‐positive CU, and MCI (Figure 3A) and were higher in amyloid PET‐positive than PET‐negative participants (Figure 3B). In same‐patient analyses, plasma miR‐214‐3p was inversely correlated with iMG Aβ42 phagocytic capacity and NCKAP1 expression (Figure 3C,D). Higher plasma miR‐214‐3p levels were also associated with worse clinical status, including lower MMSE scores and higher CDR and CDR‐SB scores (Figure 3E), and were positively correlated with plasma p‐tau217, GFAP, and NfL concentrations (Figure 3F). Together, these findings provide convergent patient‐level evidence linking elevated plasma miR‐214‐3p to reduced NCKAP1 expression, impaired iMG phagocytic capacity, and greater AD‐related biomarker and clinical burden, supporting its potential utility as a blood‐based correlate of microglial phagocytic exhaustion.

**FIGURE 3:**
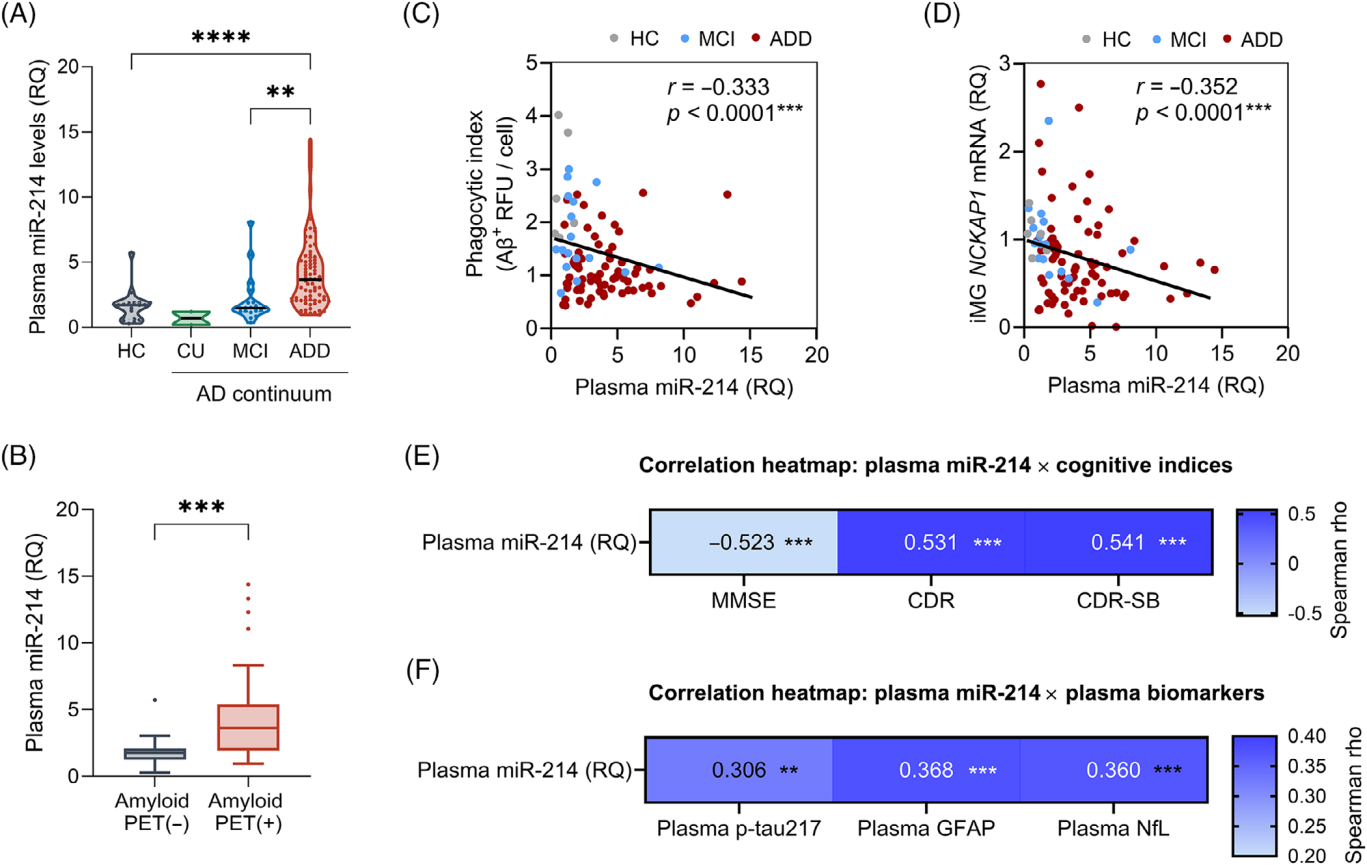
Plasma miR‐214‐3p links circulating biomarkers to NCKAP1‐dependent microglial dysfunction. (A) Plasma miR‐214‐3p across disease stages (gray = HC, green* =* amyloid‐positive CU, blue = MCI, red = ADD). (B) Plasma miR‐214‐3p levels in amyloid PET‐negative (*n =* 15) and PET‐positive (*n =* 60) participants. (C) Association between plasma miR‐214‐3p and iMG Aβ42 phagocytic capacity. (D) Association between plasma miR‐214‐3p and iMG NCKAP1 expression. (E) Correlation heatmap showing associations between plasma miR‐214‐3p and clinical measures (MMSE, CDR, and CDR‐SB). (F) Correlation heatmap showing associations between plasma miR‐214‐3p and plasma biomarkers (p‐tau217, GFAP, and NfL). For scatter plots, each point represents one participant and lines indicate linear regression fits. Statistical significance: *p < 0.05, **p < 0.01, ***p < 0.001, ****p < 0.0001. ADD, amyloid‐positive Alzheimer's disease dementia; Aβ42, amyloid beta 42; CDR, Clinical Dementia Rating; CDR‐SB, CDR Sum of Boxes; CU, amyloid‐positive cognitively unimpaired; GFAP, glial fibrillary acidic protein; HC, healthy control (amyloid‐negative cognitively unimpaired); iMG, induced microglia‐like cell; IQR, interquartile range; MCI, amyloid‐positive mild cognitive impairment; miR‐214‐3p, microRNA‐214‐3p; MMSE, Mini‐Mental State Examination; NCKAP1, non‐catalytic region of tyrosine kinase‐associated protein 1; NfL, neurofilament light chain; p‐tau217, phosphorylated tau 217; PET, positron emission tomography; RFU, relative fluorescence units; RQ, relative quantification; WRC, WAVE regulatory complex.

### 3.4 Baseline microglial markers distinguish disease severity from future progression

#### 3.4.1 Baseline NCKAP1 reflects current disease severity

We next examined whether baseline microglial measures predicted subsequent clinical progression. Among 102 participants with longitudinal follow‐up (mean 3.21 years), higher baseline iMG NCKAP1 expression was associated with slower annualized MMSE decline and less CDR‐SB worsening (Figure 4A,B). Baseline iMG phagocytic indices were also associated with subsequent progression, although generally more weakly and less consistently across cognitive measures (Figure 4E).

**FIGURE 4:**
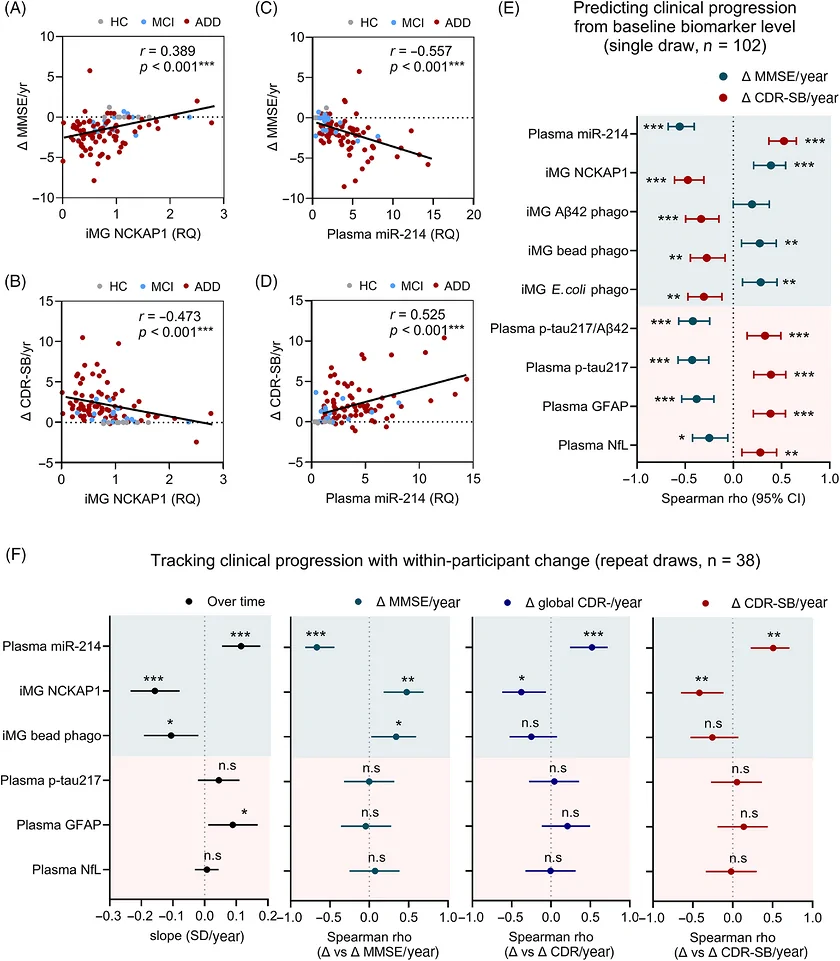
Baseline microglial and plasma markers predict clinical progression, and longitudinal microglial dynamics track disease tempo. (A–D) Associations between baseline biomarker levels and annualized clinical change in longitudinal cohort (*n =* 102): (A) iMG NCKAP1 versus ΔMMSE/year, (B) iMG NCKAP1 versus ΔCDR‐SB/year, (C) plasma miR‐214‐3p versus ΔMMSE/year, and (D) plasma miR‐214‐3p versus ΔCDR‐SB/year. Each point represents one participant and colors indicate diagnostic group (gray = HC, blue = MCI, red = ADD). Solid lines indicate linear regression fits and dotted lines indicate no annualized change. (E) Forest plot summarizing Spearman correlation coefficients (ρ, 95% CI) between baseline biomarkers and annualized clinical progression, indexed by ΔMMSE/year (teal) and ΔCDR‐SB/year (red) (*n =* 102). Shaded bands distinguish microglial (upper) and plasma (lower) biomarkers. (F) Longitudinal analyses in participants with repeat blood sampling (*n =* 38). Left, annualized within‐participant biomarker change (SD/year). Middle and right: associations between annualized biomarker change and annualized change in MMSE (teal), global CDR (blue), and CDR‐SB (red). Points represent effect estimates and 95% confidence intervals. The amyloid‐positive CU reference group was excluded from correlation analyses. Statistical significance: *p < 0.05, **p < 0.01, ***p < 0.001; ns, not significant. ADD, Alzheimer's disease dementia; CDR‐SB, Clinical Dementia Rating Sum of Boxes; CI, confidence interval; GFAP, glial fibrillary acidic protein; HC, healthy control; iMG, induced microglia‐like cell; MCI, mild cognitive impairment; MMSE, Mini‐Mental State Examination; NfL, neurofilament light chain; RQ, relative quantification.

However, these associations were markedly attenuated after adjustment for baseline cognition and were no longer significant within the amyloid‐positive ADD subgroup (Table S2), indicating that baseline NCKAP1 primarily reflects current disease severity rather than stage‐independent prognosis. Consistent with this interpretation, baseline NCKAP1 was strongly associated with contemporaneous cognitive performance.

#### 3.4.2 Plasma miR‐214‐3p provides stage‐independent prognostic information beyond conventional plasma biomarkers

In contrast, plasma miR‐214‐3p showed robust associations with subsequent clinical progression. Higher baseline plasma miR‐214‐3p predicted faster annualized MMSE decline and greater CDR‐SB worsening (Figure 4C,D). These associations remained significant after adjustment for age, baseline cognition, plasma p‐tau217, and GFAP (Table S2), indicating prognostic information beyond disease stage and conventional plasma biomarkers. Spearman correlations between baseline biomarker levels and annualized clinical change across all markers are summarized in Figure 4E.

Within amyloid‐positive ADD, participants with higher plasma miR‐214‐3p was associated with significantly faster clinical decline despite comparable baseline plasma biomarker levels and similar iMG phagocytic indices (Table S3). Incremental predictive analyses further demonstrated that plasma miR‐214‐3p significantly improved the prediction of longitudinal MMSE decline beyond conventional plasma biomarkers, whereas baseline NCKAP1 contributed only modest additional predictive value (Table S4).

Exploratory analyses of amyloid‐positive MCI participants suggested that those who later progressed to dementia had baseline biomarker profiles directionally consistent with greater disease progression, although no individual biomarker reached statistical significance because of the limited sample size (Table S5).^49^

To further evaluate the clinical utility of plasma miR‐214‐3p, participants with amyloid‐positive ADD were stratified according to plasma miR‐214‐3p tertiles (Figure 5A,B). The annualized rate of MMSE decline and the prevalence of rpAD increased progressively across tertiles. Plasma miR‐214‐3p discriminated rapid progressors substantially better than baseline NCKAP1, iMG phagocytic measures, or conventional plasma biomarkers (Figure 5C). In hierarchical regression, adding conventional plasma biomarkers to age and baseline cognition did not significantly increase the explained variance in annualized MMSE decline (Δ*R* ^2^ = 0.04, *p *= 0.35), whereas plasma miR‐214‐3p contributed substantial incremental information (Δ*R* ^2^ = 0.19, *p *< 0.001). Plasma miR‐214‐3p also improved discrimination of rapid progressors and was the only marker whose predictive performance remained robust on leave‐one‐out cross‐validation (Figure 5D), showing the strongest independent association with subsequent decline after adjustment for age, baseline MMSE, and the other plasma markers (Figure 5E), supporting its role as an independent prognostic marker.

**FIGURE 5:**
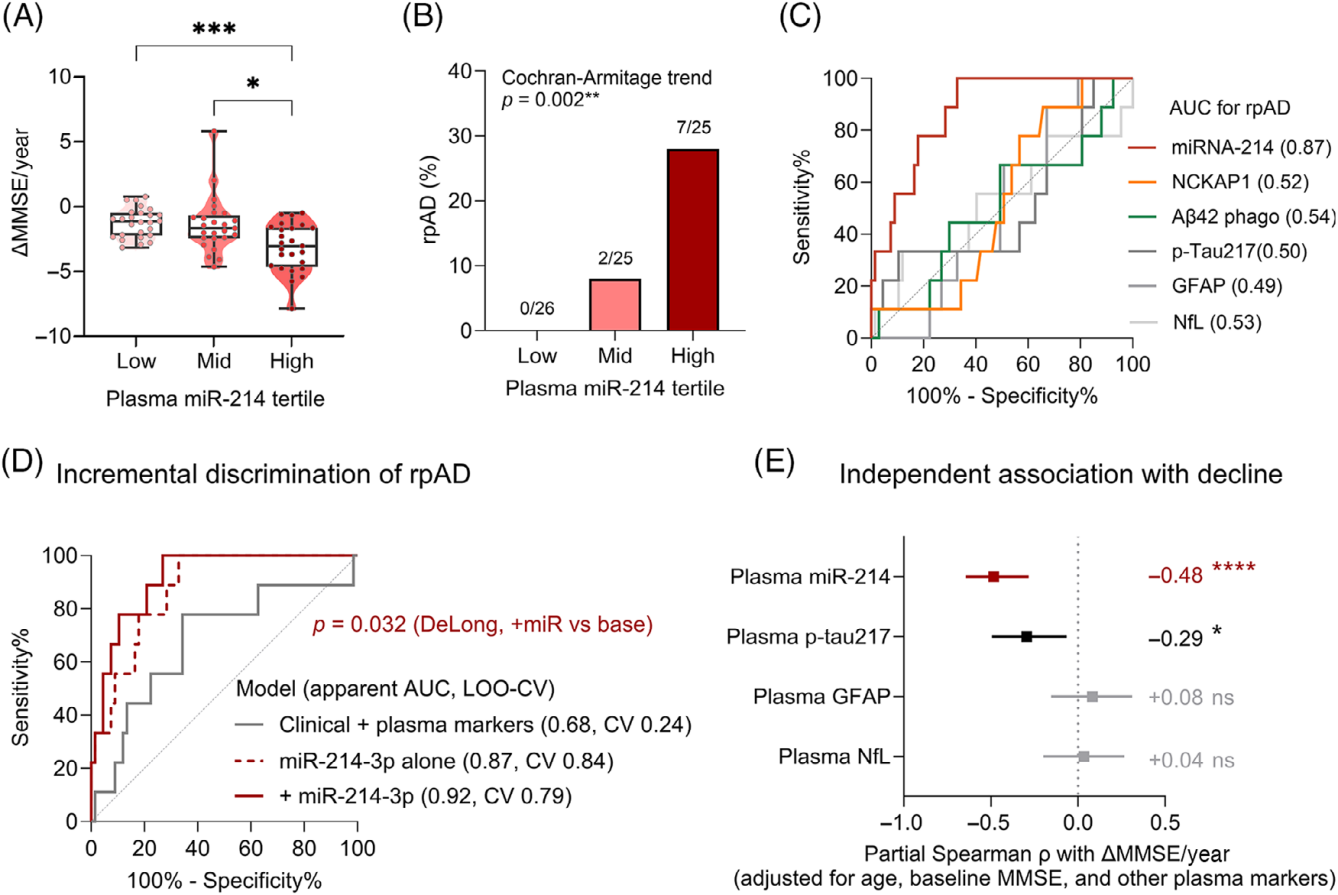
Plasma miR‐214‐3p predicts rapid cognitive progression beyond conventional plasma biomarkers. Analyses were restricted to the amyloid‐positive AD dementia subgroup (A+ADD, *n =* 76). (A) Annualized MMSE change (ΔMMSE/year) according to plasma miR‐214‐3p tertiles (low, middle, and high). (B) Frequency of rapidly progressive AD (rpAD; annualized MMSE decline ≥4 points and annualized CDR‐SB increase ≥2.5 points) across plasma miR‐214‐3p tertiles. (C) Receiver operating characteristic (ROC) curves for discrimination of rpAD using baseline biomarkers. Plasma miR‐214‐3p showed the highest discrimination (AUC = 0.87). (D) Nested ROC analysis comparing a base model (age, baseline MMSE, plasma p‐tau217, GFAP, and NfL) with models incorporating plasma miR‐214‐3p. Apparent and leave‐one‐out cross‐validated AUCs are shown. (E) Forest plot of partial Spearman correlations between plasma biomarkers and annualized MMSE decline (ΔMMSE/year), adjusted for age, baseline MMSE, and the remaining plasma biomarkers. Squares indicate partial correlation coefficients (ρ) and whiskers denote 95% confidence intervals. Group comparisons were performed using the Kruskal–Wallis test with Dunn–Bonferroni post hoc correction (A), the Cochran–Armitage trend test (B), the DeLong test for ROC comparisons (C and D), and partial Spearman correlation (E). *p < 0.05, **p < 0.01, ***p < 0.001. A+ADD, amyloid‐positive Alzheimer's disease dementia; AUC, area under the curve; CDR‐SB, Clinical Dementia Rating Sum of Boxes; GFAP, glial fibrillary acidic protein; MMSE, Mini‐Mental State Examination; NCKAP1, non‐catalytic region of tyrosine kinase‐associated protein 1; NfL, neurofilament light chain; ROC, receiver operating characteristic; rpAD, rapidly progressive AD.

### 3.5 Longitudinal microglial dynamics track disease tempo

To determine whether these baseline prognostic associations were reflected in within‐participant disease dynamics, we analyzed paired baseline and follow‐up samples from a longitudinal biomarker cohort comprising 38 participants across the amyloid‐positive continuum (median follow‐up, 3.3 years). Baseline peripheral blood mononuclear cells had been cryopreserved and processed in parallel with the corresponding follow‐up samples to minimize batch effects.

Within participants, iMG NCKAP1 expression declined over time, plasma miR‐214‐3p increased, and bead phagocytic capacity decreased, indicating progressive deterioration of microglial function (Figure S3A,B, Table S6). Conventional plasma biomarkers (p‐tau217, GFAP, and NfL) also changed longitudinally during follow‐up (Table S6).

We next examined whether the rate of biomarker change reflected the concurrent rate of clinical progression. Annualized declines in NCKAP1 and increases in plasma miR‐214‐3p were significantly associated with annualized MMSE decline and CDR‐SB worsening, whereas longitudinal changes in p‐tau217, GFAP, and NfL were not (Figure 4F; Figure S3D–F). Thus, although both microglial and conventional plasma biomarkers changed over time, only microglial dynamics tracked the tempo of clinical deterioration. Together, these longitudinal findings provide within‐participant validation of the baseline observations, demonstrating that dynamic changes in microglial markers more closely reflect disease tempo than conventional plasma biomarkers.

### 3.6 APOE ε4 modifies the trajectory of microglial phagocytic exhaustion across AD stages

We next examined whether APOE ε4 status influenced the trajectory of microglial phagocytic dysfunction. Within amyloid‐positive MCI, APOE ε4 carriers showed higher iMG Aβ42 and bead phagocytic capacity than non‐carriers (Figure S4A,B), whereas *E. coli* phagocytosis and NCKAP1 expression did not differ. By the dementia stage, carriers and non‐carriers converged to similarly low levels, reflecting a steeper decline in carriers (Aβ42, −55%; bead, −42%). This pattern suggests that APOE ε4 shapes the temporal trajectory of phagocytic exhaustion – higher MCI‐stage activity followed by a steeper decline – rather than its final extent. Given the small APOE‐stratified subgroups, these observations are exploratory.

Although formal interaction testing was not significant, the inverse association between plasma p‐tau217 and iMG phagocytic function appeared more evident in APOE ε4 non‐carriers, whereas plasma GFAP associations were seen in both groups (Figure S4C–F). These stratified analyses should be interpreted cautiously given the limited subgroup sizes.

### 3.7 Autophagic‐lysosomal stress signatures and CSF correlates of microglial dysfunction

To characterize the molecular context of iMG phagocytic dysfunction, we examined autophagic‐lysosomal markers. Rubicon, a regulator of LC3‐associated phagocytosis and autophagic flux,^50^ showed a biphasic pattern, peaking at MCI before declining in ADD, whereas EIF5A and COX2 decreased progressively across the continuum (Figure S5A–C). Rubicon correlated with TFEB, TFE3, and LAMP1 (Figure S5D–F), consistent with coordinated lysosomal stress responses, and EIF5A was positively associated with Aβ42 phagocytic capacity and NCKAP1 expression (Figure S5G,H).

To assess whether these peripheral signatures were mirrored in the central nervous system (CNS), we examined CSF biomarkers in a subset (*n =* 54). CSF soluble TREM2 increased and TGF‐β1 declined across disease stages (Figure S6G,I). The CSF Aβ42/40 ratio was positively associated with iMG Aβ42 phagocytic capacity, whereas CSF p‐tau181 and IL‐10 showed inverse associations (Figure S5I–K), providing CNS‐compartment support for the link between amyloid‐related biomarker burden and patient‐specific microglial dysfunction. Full CSF profiles are shown in Figure S6.

## 4 DISCUSSION

In this same‐patient study of healthy controls (HC) and individuals across the amyloid‐positive AD continuum, we integrated PBMC‐derived iMG functional profiles with plasma and CSF biomarkers, amyloid imaging, APOE ε4 status, and longitudinal clinical outcomes. The principal finding is that patient‐specific microglial dysfunction provides information that complements, rather than duplicates, conventional plasma biomarkers. iMG phagocytic capacity progressively declined across the AD continuum, accompanied by reduced NCKAP1 expression and stage‐dependent autophagic‐lysosomal stress responses. Baseline NCKAP1 primarily reflected current disease severity, whereas plasma miR‐214‐3p identified individuals with faster subsequent decline despite comparable conventional plasma biomarker burden. Importantly, longitudinal analyses further demonstrated that within‐participant microglial dynamics – declining NCKAP1 and rising plasma miR‐214‐3p – but not conventional plasma biomarkers, tracked the tempo of cognitive decline. Together, these findings suggest that microglial dysfunction contributes to biological‐clinical discordance and provides a complementary functional dimension of AD progression.

The 2024 revised AD framework emphasizes that biological staging should incorporate not only amyloid, tau, and neurodegeneration but also inflammatory and immune processes, co‐pathologies, and biological‐clinical staging.^16^ This is particularly relevant because biomarker‐defined AD pathology does not always explain clinical severity or clinical trajectory. Our findings support the concept that microglial functional status may be one contributor to this discordance.

Conventional plasma biomarkers such as p‐tau217, GFAP, and NfL provide powerful information about AD pathology, astroglial activation, and neuronal injury, but they do not directly measure microglial functional capacity. By contrast, iMG‐derived phagocytic profiles provide a patient‐level functional readout of immune‐inflammatory activity across disease stages. Among conventional plasma biomarkers, GFAP showed the strongest association with iMG phagocytic dysfunction, consistent with the close interaction between astrocytes and microglia during AD neuroinflammation.^51^, ^52^ However, GFAP should not be interpreted as a direct measure of microglial function. Rather, it appears to reflect a glial response that parallels, but does not directly capture, microglial functional status. These observations support microglial dysfunction as a complementary functional layer of disease biology beyond conventional plasma biomarkers.

NCKAP1 provided a molecular link between iMG phagocytic dysfunction and clinical status. As a component of the WAVE regulatory complex, NCKAP1 is required for actin remodeling and phagocytic cup formation.^14^, ^30^ Consistent with our previous work,^7^ lower NCKAP1 expression was associated with reduced phagocytic capacity, worse cognition, and progressive decline across the AD continuum, in agreement with reports of reduced NCKAP1 expression in AD brain tissue.^53^, ^54^ However, after adjustment for baseline cognition, its association with longitudinal progression was largely attenuated, indicating that baseline NCKAP1 primarily reflects the current functional state of microglia and disease severity rather than stage‐independent prognosis. Nevertheless, longitudinal within‐participant analyses demonstrated that declining NCKAP1 tracked concurrent cognitive worsening, suggesting that temporal changes in NCKAP1 may provide more informative measures of disease progression than a single baseline assessment.

In contrast, plasma miR‐214‐3p emerged as a clinically accessible marker of disease progression. Higher plasma miR‐214‐3p levels were associated with faster cognitive decline, greater CDR‐SB worsening, and enrichment of patients with rpAD despite comparable conventional plasma biomarker profiles and similar baseline iMG phagocytic measures. These associations remained significant after adjustment for baseline cognition, p‐tau217, and GFAP, indicating that miR‐214‐3p captures a progression‐related dimension not explained by disease stage or conventional biomarker burden. Given its predicted regulation of NCKAP1 and its previously reported role in impaired microglial phagocytosis,^7^, ^11^ plasma miR‐214‐3p may provide an accessible blood‐based marker for refining prognosis beyond conventional biomarker staging. Similar observations in ALS and prodromal PD further suggest that miR‐214‐3p participates in shared neurodegeneration‐associated stress responses.^8^, ^55^, ^56^ Together, these findings suggest that plasma miR‐214‐3p captures a progression‐related (disease‐tempo) dimension that complements, rather than duplicates, information provided by conventional plasma biomarkers.

APOE ε4 carriers exhibited higher phagocytic activity at the MCI stage followed by a steeper decline toward ADD, consistent with stage‐dependent differences in microglial functional adaptation. Although these findings should be interpreted cautiously because of limited subgroup sizes, they are consistent with growing evidence that APOE ε4 modifies microglial responses during AD progression.^27^, ^28^ Rather than implying a direct causal effect, these findings suggest that APOE ε4 may modulate the trajectory of microglial functional adaptation across disease stages.

The contribution of biological aging also warrants consideration. Age was associated with iMG phagocytic capacity and with plasma GFAP and NfL (Table S7), consistent with links among aging, inflammaging, and microglial senescence.^57^, ^58^ However, age‐adjusted analyses demonstrated that phagocytic dysfunction, NCKAP1 reduction, and the GFAP‐phagocytosis relationship persisted beyond the effects of aging alone. Furthermore, neither NCKAP1 nor plasma miR‐214‐3p was significantly associated with age. These findings support a distinction between age‐related microglial senescence and disease‐associated phagocytic exhaustion linked to AD pathology and APOE ε4‐related vulnerability.^59^, ^60^

Current AD trials increasingly rely on amyloid and tau biomarkers for enrollment and staging, but clinical progression and treatment response remain heterogeneous. Patient‐derived microglial dysfunction profiles may help identify individuals whose clinical trajectory is not fully explained by conventional biomarker burden. Because longitudinal microglial dynamics tracked disease tempo more closely than conventional plasma biomarkers, this approach may be particularly valuable for longitudinal monitoring and for trials targeting microglial or immune pathways. Although iMG profiling is not yet ready for routine clinical application, it provides a testable framework for biologically informed patient stratification.

This study has several limitations. First, the amyloid‐positive CU and MCI groups were small, limiting interpretation of early‐stage changes, particularly the APOE ε4‐stratified analyses and the MCI‐stage Rubicon response. Second, objective amyloid confirmation by PET or CSF was not available for all participants; plasma p‐tau217 was used only as a supportive classifier when PET or CSF data were unavailable. Third, although iMGs are generated from peripheral monocytes and cannot fully reproduce the ontogeny, tissue imprinting, or microenvironmental context of CNS‐resident microglia, the differentiation protocol used here has been shown to recapitulate key transcriptomic, phenotypic, and functional features of human microglia,^7^, ^37^, ^38^ supporting its use as a patient‐specific assay of microglial functional capacity rather than a surrogate for in situ brain microglia. Concordant CSF findings – including increased soluble TREM2, decreased TGF‐β1, and positive associations between CSF Aβ42/40 and iMG phagocytic capacity – further support the biological relevance of these peripheral iMG signatures to CNS microglial function. Fourth, the observational design limits causal interpretation of the relationship between microglial dysfunction and clinical progression. Although the longitudinal cohort (*n =* 38; median follow‐up 3.3 years) remained modest in size and the follow‐up interval was heterogeneous, it provided independent within‐participant support for the cross‐sectional findings, demonstrating that declining NCKAP1 and rising plasma miR‐214‐3p tracked concurrent cognitive deterioration. Because annualized rates assume approximately linear within‐participant change, these findings should be confirmed in larger cohorts with standardized longitudinal sampling. Finally, the prognostic role of plasma miR‐214‐3p and the proposed stratification framework require validation in independent, prospectively staged cohorts. In particular, the rapid‐progression analyses were based on only nine rapid progressors among 76 participants with ADD; therefore, the associated discrimination, cut‐off, and incremental predictive estimates should be regarded as hypothesis‐generating until externally validated.

In conclusion, same‐patient profiling identified progressive microglial phagocytic dysfunction as a complementary functional dimension of AD progression. Baseline NCKAP1 primarily reflected current disease severity, whereas longitudinal microglial dynamics – including declining NCKAP1 and rising plasma miR‐214‐3p – tracked disease tempo more closely than conventional plasma biomarkers. Together, miR‐214‐3p and NCKAP1 provide complementary readouts of microglial dysfunction and disease dynamics, supporting a microglial functional dimension of AD progression beyond conventional measures of pathological burden. Patient‐derived microglial profiling may therefore provide an adjunctive approach for interpreting biological‐clinical discordance and help operationalize the neuroinflammatory “I” component at the cellular level within future biologically informed staging frameworks. Beyond their biomarker relevance, the NCKAP1‐phagocytosis axis and its putative regulator miR‐214‐3p may also represent potential therapeutic entry points, although the regulatory specificity of miR‐214‐3p and the causal role of this pathway in AD require dedicated mechanistic validation.

## CONFLICT OF INTEREST STATEMENT

The authors declare no conflicts of interest. Author disclosures are available in the Supporting Information.

## CONSENT STATEMENT

Written informed consent was obtained from all participants.

## Supporting information




**Supporting information**: alz71903‐sup‐0001‐ICMJE.pdf

**Supporting information**: alz71903‐sup‐0002‐SuppMat.docx
