# Supplementary material for: miR‐214‐3p and NCKAP1: A microglial functional dimension of Alzheimer's progression
Source: Alzheimers Dement. 2026 Oct 7;22(10):e71903. doi: 10.1002/alz.71903 (PMC13643821; doi:10.1002/alz.71903)
Supplement: Supplementary file 2 [file ALZ-22-e71903-s001.docx]

**Supplemental Online content**

**Supplementary Methods**

**Figure S1.** Performance of plasma biomarkers for amyloid PET positivity and their associations with clinical and pathological measures.

**Figure S2.** Sensitivity analysis including amyloid-negative cognitively impaired participants.

**Figure S3.** Longitudinal within-participant changes in microglial markers and their association with cognitive decline.

**Figure S4.** APOE ε4-stratified microglial phagocytic trajectories and plasma–microglial associations (exploratory).

**Figure S5.** Autophagic–lysosomal stress signatures and CSF correlates of microglial dysfunction.

**Figure S6.** Cerebrospinal fluid biomarker profiles across the AD continuum in the CSF sub-cohort.

**Table S1.** Technical Details of Biomarker Measurements, Phagocytosis Assays, and Gene Expression Analyses

**Table S2.** Sensitivity of baseline marker–progression associations to adjustment for baseline cognition.

**Table S3.** Clinical and biomarker characteristics according to microglial phagocytic dysfunction status in amyloid-positive AD dementia.

**Table S4.** Incremental prognostic performance of iMG measures beyond the clinical/biomarker base model.

**Table S5.** Baseline iMG and plasma biomarker profiles of amyloid-positive MCI participants who converted to dementia versus non-converters.

**Table S6.** Longitudinal changes in iMG and plasma markers and their associations with cognitive decline.

**Table S7.** Age associations and age-adjusted analyses of microglial markers.

**Supplementary Methods**

**APOE Genotyping**

Genomic DNA was isolated using the QIAsymphony DSP DNA Mini Kit (Qiagen, Hilden, Germany) on an automated QIAsymphony SP platform according to the manufacturer's instructions. APOE genotyping was performed using the Real-Q APOE Genotyping Kit (Biosewoom, Seoul, Korea) on a CFX96 Real-Time PCR Detection System (Bio-Rad, Hercules, CA, USA).

**Generation of Human iMGs**

PBMCs were isolated by Ficoll density-gradient centrifugation and cultured overnight in RPMI-1640 supplemented with 10% fetal bovine serum and 1% antibiotic/antimycotic solution. Adherent monocytes were subsequently differentiated into iMGs using RPMI-1640 Glutamax supplemented with recombinant human GM-CSF and IL-34 for 14 days at 37°C in a humidified atmosphere containing 5% CO₂.

**iMG Phagocytosis Assays**

Phagocytic activity was evaluated using fluorescent latex beads (Sigma-Aldrich), fluorescently labeled Aβ42 fibrils (Anaspec), and pHrodo-conjugated E. coli bioparticles (Invitrogen). Cells were incubated with substrates for 2 h at 37°C, washed with PBS, fixed, and stained with Alexa Fluor 488-conjugated phalloidin. Images were acquired using a Leica TCS SP5 confocal microscope and analyzed using ImageJ software. Phagocytic uptake was quantified as beads per cell, Aβ42 fluorescence intensity per cell, or E. coli uptake per cell.

**Quantitative Real-Time PCR**

Total RNA was extracted using TRIzol reagent and reverse transcribed using a High-Capacity cDNA Reverse Transcription Kit. Quantitative PCR was performed using SYBR Green chemistry according to the manufacturers’ instructions. Gene expression levels were normalized to GAPDH and expressed as relative quantification values.

**Statistical Analysis**

Correlation and Adjustment Analyses

Associations between iMG-derived measures, plasma biomarkers, CSF biomarkers, and clinical variables were assessed using Spearman rank correlation. To evaluate whether associations between NCKAP1 expression and longitudinal clinical progression were independent of established plasma biomarkers, partial correlations were calculated after regressing both variables on plasma p-tau217 and GFAP and correlating the resulting residuals. To assess age-independence of key findings, age-adjusted analyses were performed using residuals derived from linear regression models with age as a covariate. Group differences were re-evaluated using age-residualized values, and selected correlations were repeated using age-adjusted partial correlation analyses.

APOE ε4 Stratification Analyses

APOE ε4 subgroup analyses were performed to explore potential genotype-dependent differences in the relationship between plasma biomarkers and iMG functional measures. Correlation coefficients between APOE ε4 carriers and non-carriers were compared using Fisher’s *r*-to-*z* transformation. Within-stage comparisons were evaluated using the Mann–Whitney U test. Potential effect modification by APOE ε4 status was assessed by including a multiplicative interaction term (plasma p-tau217 × APOE ε4 status) in regression models.

Longitudinal Progression Analyses

Associations between baseline biomarkers and subsequent clinical progression were evaluated using ordinary least-squares regression models with annualized MMSE change (ΔMMSE/year) or annualized CDR-SB change (ΔCDR-SB/year) as outcome variables. Models were adjusted for age and baseline cognitive performance, with additional analyses further adjusting for plasma p-tau217 and/or GFAP where appropriate.

For the longitudinal biomarker cohort, baseline and follow-up values were compared using the Wilcoxon signed-rank test. Within-participant biomarker change was expressed as both absolute change and annualized change (Δ/year), calculated by dividing the difference between follow-up and baseline values by the follow-up interval. Associations between annualized biomarker change and annualized clinical change were assessed using Spearman correlation. Population-level longitudinal trajectories were additionally estimated using linear mixed-effects models with time (years from baseline) as a fixed effect and participant as a random intercept. Right-skewed variables were log-transformed where appropriate, and biomarkers were standardized before model fitting.

Rapid Progression Analyses

Rapidly progressive AD (rpAD) was defined a priori as concurrent annualized MMSE decline ≥4 points and annualized CDR-SB increase ≥2.5 points. Participants with amyloid-positive AD dementia were additionally stratified into tertiles according to baseline plasma miR-214-3p expression. Differences in annualized MMSE decline across tertiles were evaluated using the Kruskal–Wallis test with Dunn–Bonferroni post-hoc comparisons, and trends in rpAD prevalence were assessed using the Cochran–Armitage trend test. Because complete separation occurred in the lowest miR-214-3p subgroup, comparisons of rpAD frequency were performed using Fisher's exact test rather than adjusted logistic regression.

ROC Analyses

Receiver operating characteristic (ROC) analyses were performed to evaluate the ability of baseline biomarkers to discriminate rpAD. Areas under the ROC curve (AUCs) were compared using DeLong's test for correlated ROC curves. Optimal cut-off values were determined using the Youden index and are presented for descriptive purposes only because of the limited number of rapidly progressive cases.

Incremental Predictive Performance Analyses

To determine whether iMG-derived measures provided prognostic information beyond conventional plasma biomarkers, nested models were constructed using age, log-transformed plasma p-tau217, and plasma GFAP as the reference model. For longitudinal outcomes, baseline cognitive performance was additionally included as a covariate. Augmented models incorporated one iMG-derived measure at a time, including NCKAP1 expression, plasma miR-214-3p, or the phagocytic composite score. The phagocytic composite score was calculated as the standardized mean of the z-scored Aβ42, bead, and E. coli phagocytic indices.

Model discrimination was evaluated using Harrell's C statistic. Incremental predictive value (ΔC) was defined as the difference between augmented and reference models. Bootstrap resampling (1,000 iterations) was used to estimate optimism-corrected C statistics, 95% confidence intervals, and incremental discrimination statistics. Model improvement was additionally assessed using likelihood-ratio tests and changes in adjusted R². Because no independent validation cohort was available, these analyses should be considered exploratory and internally validated only.

**Figure S1. Performance of plasma biomarkers for amyloid PET positivity and their associations with clinical and pathological measures.**

**(A–E)** Plasma biomarker levels in amyloid PET-negative (n = 18) and amyloid PET-positive (n = 57) participants: **(A)** p-tau217, **(B)** p-tau217/Aβ42 ratio, **(C)** Aβ42/40 ratio, **(D)** GFAP, and **(E)** NfL. p-tau217, the p-tau217/Aβ42 ratio, GFAP, and NfL were higher, whereas the Aβ42/40 ratio was lower, in PET-positive participants. Boxes indicate the median and interquartile range (IQR), whiskers extend to 1.5×IQR, and outliers are shown individually. Between-group differences were assessed using the Mann–Whitney U test. Statistical significance: ****p*<0.001, *****p*<0.0001. **(F)** Receiver operating characteristic (ROC) curves for discriminating amyloid PET positivity using each plasma biomarker. The p-tau217/Aβ42 ratio showed the highest diagnostic performance (AUC=0.937). **(G)** Diagnostic performance of each plasma biomarker for amyloid PET positivity, including AUC (95% CI), optimal cutoff, sensitivity, and specificity. The p-tau217 cutoff of 0.509 pg/mL corresponds to the threshold applied throughout this study. **(H)** Spearman correlation matrix among plasma biomarkers, cognitive measures (MMSE, CDR, CDR-SB), age, amyloid PET status, and APOE ε4 carrier status. Cell color and displayed coefficients indicate the direction and strength of correlations (blue, positive; red, negative). Plasma p-tau217, the p-tau217/Aβ42 ratio, GFAP, and NfL showed expected associations with amyloid PET status and clinical measures.

*Abbreviations*: APOE ε4, apolipoprotein E ε4; AUC, area under the curve; Aβ40, amyloid-β 40; Aβ42, amyloid-β 42; Aβ42/40, amyloid-β 42/40 ratio; CDR, Clinical Dementia Rating; CDR-SB, CDR Sum of Boxes; CI, confidence interval; GFAP, glial fibrillary acidic protein; IQR, interquartile range; MMSE, Mini-Mental State Examination; NfL, neurofilament light chain; p-tau217, phosphorylated tau 217; PET, positron emission tomography; ROC, receiver operating characteristic.

**Figure S2. Sensitivity analysis including amyloid-negative cognitively impaired participants.**

Microglial and plasma biomarkers across the full participant spectrum: amyloid-negative cognitively unimpaired controls (A− CU [HC], n=18), the amyloid-positive AD continuum (A+ CU, n=2; A+ MCI, n=18; A+ ADD, n=76), and amyloid-negative cognitively impaired participants (A− MCI, n=4; A− AD, n=10). The amyloid-negative cognitively impaired groups were excluded from the primary analyses and are included here as a sensitivity analysis. (A) Aβ42 phagocytic index, (B) bead phagocytic index, (C) E. coli phagocytic index, and (D) iMG NCKAP1 expression, (E) plasma miR-214-3p, (F) plasma GFAP, (G) plasma p-tau217, and (H) plasma NfL. Violin plots show individual participants with medians indicated. The A+ CU group (n=2) is shown for descriptive reference only and was not included in statistical testing. Group comparisons were performed using the Kruskal–Wallis test with Dunn–Bonferroni post-hoc correction. Only significant pairwise comparisons are indicated (**p*<0.05, ***p*<0.01, ****p*<0.001, *****p*<0.0001). The overall patterns of reduced iMG phagocytic capacity and NCKAP1 expression, together with increased plasma miR-214-3p, were preserved when amyloid-negative cognitively impaired participants were included.

*Abbreviations*: ADD, amyloid-positive Alzheimer’s disease dementia; Aβ42, amyloid-β 42; CU, amyloid-positive cognitively unimpaired; HC, healthy control (amyloid-negative cognitively unimpaired); iMG, induced microglia-like cell; IQR, interquartile range; MCI, amyloid-positive mild cognitive impairment; NCKAP1, NCK-associated protein 1; RFU, relative fluorescence units; RQ, relative quantification.

**Figure S3. Longitudinal within-participant changes in microglial markers and their association with cognitive decline.**

Paired baseline and follow-up measurements were available in a longitudinal subset (n=38; HC n=3, amyloid-positive MCI n=4, ADD n=31). **(A–C)** Within-participant trajectories for **(A)** iMG NCKAP1 expression, **(B)** plasma miR-214-3p, and **(C)** iMG bead phagocytosis. Thin lines connect baseline and follow-up measurements for each participant; bold black lines indicate median trajectories. Colors indicate baseline diagnostic group (grey=HC, blue=MCI, red=ADD). P values were calculated using the Wilcoxon signed-rank test. **(D–F)** Associations between annualized biomarker change and annualized cognitive change (ΔMMSE/year): **(D)** ΔNCKAP1/year, **(E)** ΔmiR-214-3p/year, and **(F)** Δbead phagocytosis/year. Lines indicate ordinary least-squares regression fits. Correlation coefficients (*r*) and *p* values are shown in the panels. Negative ΔMMSE/year values indicate cognitive decline.

*Abbreviations*: ADD, amyloid-positive Alzheimer's disease dementia; HC, healthy control (amyloid-negative cognitively unimpaired); iMG, induced microglia-like cell; MCI, amyloid-positive mild cognitive impairment; miR-214-3p, microRNA-214-3p; MMSE, Mini-Mental State Examination; NCKAP1, NCK-associated protein 1; RQ, relative quantification.

**Figure S4. APOE ε4-stratified microglial phagocytic trajectories and plasma–microglial associations (exploratory).**

**(A)** iMG Aβ42 phagocytic index and **(B)** bead phagocytic index according to APOE ε4 status across disease stages (HC, amyloid-positive CU, MCI, and ADD). APOE ε4 analyses included amyloid-classified participants with available genotyping (n=98; ε4+ n=45, ε4− n=53). Because the HC and amyloid-positive CU subgroups contained very small numbers of APOE-stratified participants, these groups are shown for descriptive reference only and were not formally compared. Comparisons between ε4 carriers and non-carriers were restricted to the MCI and ADD stages. **(C)** Plasma p-tau217 levels according to APOE ε4 status. **(D)** Association between plasma p-tau217 and iMG Aβ42 phagocytic capacity stratified by APOE ε4 status. **(E)** Association between plasma p-tau217 and iMG bead phagocytic capacity stratified by APOE ε4 status. **(F)** Forest plot showing Spearman correlation coefficients (95% CI) between plasma biomarkers (GFAP and p-tau217) and iMG functional indices (NCKAP1 expression, Aβ42 phagocytosis, and bead phagocytosis), stratified by APOE ε4 status. Box plots show median, interquartile range (IQR), and 1.5×IQR whiskers. Scatter plots display individual participants and linear regression lines. Group comparisons were performed using the Mann–Whitney U test. Correlations were assessed using Spearman rank analysis. Formal APOE ε4 interaction testing was not significant, and all APOE-stratified analyses should be considered exploratory and hypothesis-generating. Statistical significance: **p*<0.05, ***p*<0.01, ****p*<0.001, *****p*<0.0001; ns, not significant.

*Abbreviations*: ADD, amyloid-positive Alzheimer’s disease dementia; APOE ε4, apolipoprotein E ε4; Aβ42, amyloid-β 42; CI, confidence interval; CU, amyloid-positive cognitively unimpaired; GFAP, glial fibrillary acidic protein; HC, healthy control (amyloid-negative cognitively unimpaired); iMG, induced microglia-like cell; IQR, interquartile range; MCI, amyloid-positive mild cognitive impairment; NCKAP1, NCK-associated protein 1; p-tau217, phosphorylated tau 217.

**Figure S5. Autophagic–lysosomal stress signatures and CSF correlates of microglial dysfunction.**

**(A)** iMG Rubicon expression **(B)** iMG EIF5A expression. **(C)** iMG COX2 (PTGS2) expression across the AD continuum. **(D–F)** Associations between iMG Rubicon expression and **(D)** TFEB, **(E)** TFE3, and **(F)** LAMP1 expression. **(G-H)** Association between iMG EIF5A expression and **(G)** Aβ42 phagocytic capacity and **(H)** NCKAP1 expression. **(I–K)** Associations between iMG Aβ42 phagocytic capacity and **(I)** CSF Aβ42/40 ratio, **(J)** CSF p-tau181, and **(K)** CSF IL-10. For scatter plots, each point represents one participant and lines indicate linear regression fits. Data are presented as median and interquartile range (IQR) unless otherwise indicated. Statistical significance: **p*<0.05, ***p*<0.01, *****p*<0.0001.

*Abbreviations*: ADD, amyloid-positive Alzheimer’s disease dementia; Aβ42, amyloid-β 42; Aβ42/40, amyloid-β 42/40 ratio; COX2, cyclooxygenase-2; CSF, cerebrospinal fluid; CU, amyloid-positive cognitively unimpaired; EIF5A, eukaryotic translation initiation factor 5A; HC, healthy control (amyloid-negative cognitively unimpaired); iMG, induced microglia-like cell; IQR, interquartile range; LAMP1, lysosomal-associated membrane protein 1; MCI, amyloid-positive mild cognitive impairment; NCKAP1, NCK-associated protein 1; RFU, relative fluorescence units; RQ, relative quantification; Rubicon, RUN domain Beclin-1-interacting and cysteine-rich-containing protein; TFE3, transcription factor E3; TFEB, transcription factor EB.

**Figure S6. Cerebrospinal fluid biomarker profiles across the AD continuum in the CSF sub-cohort.**

CSF biomarkers were measured in participants with available CSF samples (HC, n=4; MCI, n=5; ADD, n=45; total n=54). (A) CSF Aβ42, (B) CSF Aβ42/40 ratio, (C) total tau (tTau), (D) p-tau181, (E) tau/Aβ42 ratio, (F) p-tau181/Aβ42 ratio, (G) soluble TREM2, and (H) NfL across disease stages. (I) TGF-β1, (J) IL-10, (K) IL-15, (L) IL-1RA, (M) IL-6, (N) IP-10, (O) MIP-1β, and (P) TNF-α across disease stages. Data are presented as violin plots with individual data points overlaid; horizontal lines indicate the median and interquartile range (IQR). Group comparisons were performed using the Kruskal–Wallis test with Dunn–Bonferroni post-hoc correction. Statistical significance: **p*<0.05, ***p*<0.01, ****p*<0.001, *****p*<0.0001. Because of the limited number of HC (n=4) and MCI (n=5) participants, comparisons involving these groups should be considered exploratory and hypothesis-generating.

*Abbreviations*: ADD, amyloid-positive Alzheimer’s disease dementia; Aβ42, amyloid-β 42; Aβ42/40, amyloid-β 42/40 ratio; CSF, cerebrospinal fluid; HC, healthy control (amyloid-negative cognitively unimpaired); IQR, interquartile range; MCI, amyloid-positive mild cognitive impairment; NfL, neurofilament light chain; TREM2, triggering receptor expressed on myeloid cells 2.

**Table S1. Technical Details of Biomarker Measurements, Phagocytosis Assays, and Gene Expression Analyses.**

| Category | Target / Assay | Platform / Reagent | Manufacturer | Assay ID / Catalog No. |
| --- | --- | --- | --- | --- |
| APOE Genotyping | APOE ε2/ε3/ε4 | Real-Q APOE Genotyping Kit | Biosewoom | Real-Q APOE Kit |
| Plasma Biomarker | p-tau217 | Simoa Human ALZpath p-tau217 Advantage PLUS | Quanterix | REF 104570 |
| Plasma Biomarker | GFAP | Simoa Neurology 4-Plex E Advantage PLUS | Quanterix | REF 104465 |
| Plasma Biomarker | NfL | Simoa Neurology 4-Plex E Advantage PLUS | Quanterix | REF 104465 |
| Plasma Biomarker | Aβ40 | Simoa Neurology 4-Plex E Advantage PLUS | Quanterix | REF 104465 |
| Plasma Biomarker | Aβ42 | Simoa Neurology 4-Plex E Advantage PLUS | Quanterix | REF 104465 |
| Plasma Analysis Platform | Simoa HD-X Analyzer | Digital immunoassay platform | Quanterix | — |
| CSF Biomarker | Soluble TREM2 | Human TREM2 SimpleStep ELISA | Abcam | ab224881 |
| CSF Cytokine Panel | IL-6, IL-8, IL-10, IP-10, MCP-1, TNF-α, MIP-1β | Human Cytokine Magnetic Bead Panel | Millipore | HCYTOMAG-60K-11 |
| CSF Cytokine Panel | TGF-β1 | TGF-β Magnetic Bead Panel | Millipore | TGFBMAG-64K-01 |
| CSF Biomarker | Aβ42/40, Total Tau, p-tau181 | MILLIPLEX Human Amyloid Beta and Tau Panel | Millipore | HNABTMAG-68K |
| CSF Biomarker | NfL | NF-light ELISA | UMAN Diagnostics | NF-light® ELISA |
| Phagocytosis Assay | Fluorescent Latex Beads | Bulk phagocytosis substrate | Sigma-Aldrich | L3030 |
| Phagocytosis Assay | Fluorescent Aβ42 Fibrils | Disease-relevant clearance substrate | Anaspec | — |
| Phagocytosis Assay | pHrodo E. coli Bioparticles | Innate immune phagocytosis substrate | Invitrogen | P35366 |
| miRNA Assay | hsa-miR-214-3p | miRCURY LNA PCR Assay | Qiagen | YP00204510 |
| miRNA Normalizer | hsa-miR-103-3p | miRCURY LNA PCR Assay | Qiagen | YP00204063 |
| miRNA Normalizer | hsa-miR-423-5p | miRCURY LNA PCR Assay | Qiagen | YP00205624 |
| qPCR Target | NCKAP1 | RT-qPCR Assay | Qiagen | PPH15666A |
| qPCR Target | RUBCN (Rubicon) | RT-qPCR Assay | OriGene | HP211071 |
| qPCR Target | EIF5A | RT-qPCR Assay | Qiagen | PPH01296A |
| qPCR Target | TFEB | RT-qPCR Assay | Qiagen | PPH08472A |
| qPCR Target | TFE3 | RT-qPCR Assay | Qiagen | PPH15555A |
| qPCR Target | LAMP1 | RT-qPCR Assay | Qiagen | PPH05794G |
| qPCR Target | COX2 (PTGS2) | RT-qPCR Assay | Qiagen | PPH01136F |
| Reference Gene | GAPDH | RT-qPCR Assay | Qiagen | PPH00150F |

**Table S2. Sensitivity of baseline marker–progression associations to adjustment for baseline cognition.**

**Baseline (unadjusted) associations of each marker with annualized clinical change (n=102).**

| **Baseline marker** | **ΔMMSE/year, ρ (95% CI)** | **ΔCDR-SB/year, ρ (95% CI)** | **Direction of association** |
| --- | --- | --- | --- |
| iMG Aβ42 phagocytosis | +0.193 (−0.002, 0.373), n.s. | −0.334 (−0.496, −0.149) *** | sig. for ΔCDR-SB only |
| **iMG NCKAP1** | +0.389 (0.210, 0.542) *** | −0.473 (−0.611, −0.306) *** | higher → slower decline |
| **Plasma miR-214-3p** | −0.557 (−0.678, −0.407) *** | +0.525 (0.368, 0.653) *** | higher → faster decline |
| Plasma p-tau217 | −0.428 (−0.575, −0.255) *** | +0.390 (0.212, 0.543) *** | higher → faster decline |
| Plasma GFAP | −0.381 (−0.536, −0.202) *** | +0.386 (0.207, 0.540) *** | higher → faster decline |

Spearman ρ (unadjusted). Because higher MMSE and lower CDR-SB both denote better function, a protective marker shows opposite signs across the two outcomes (positive for ΔMMSE/year, negative for ΔCDR-SB/year); the Direction column gives the clinical interpretation. All markers except iMG Aβ42 phagocytosis are significant at baseline; whether each signal survives covariate adjustment is shown in the Summary panel and Panels A–D below. ****p*<0.001, ***p*<0.01, **p*<0.05.

**Summary panel. Head-to-head sensitivity of NCKAP1 versus plasma miR-214-3p (outcome = ΔCDR-SB/year, full cohort). Bold = *p*<0.05.**

| **Baseline predictor (ΔCDR-SB/year)** | **Unadjusted** | **+ p-tau217 + GFAP** | **+ baseline MMSE** | **+ baseline MMSE + age** | **+ all covariates** |
| --- | --- | --- | --- | --- | --- |
| iMG NCKAP1 (Fig 4B) | −0.473 (<0.001) | −0.389 (<0.001) | −0.250 (0.013) | −0.241 (0.017) | −0.241 (0.017) |
| Plasma miR-214-3p (Fig 4D) | +0.525 (<0.001) | +0.449 (<0.001) | +0.335 (<0.001) | +0.332 (<0.001) | +0.332 (<0.001) |

Higher NCKAP1 and lower miR-214-3p indicate slower decline; the NCKAP1 association attenuates but remains significant after adjustment for baseline cognition, whereas the miR-214-3p association persists more strongly. NCKAP1 detail for both outcomes and the within-ADD stratum is shown in Panels A–C; the full baseline-predictor matrix is in Panel D. Corresponding ΔMMSE/year values for miR-214-3p are not yet tabulated here.

| **Panel A. Full analyzed cohort (iMG NCKAP1)** | | | | | | |
| --- | --- | --- | --- | --- | --- | --- |
| **Outcome** | **Adjustment model** | | | **n** | **Partial Spearman *r*** | ***p* value** |
| ΔMMSE/year | Unadjusted | | | 102 | +0.389 | <0.001 |
| ΔMMSE/year | Adjusted for p-tau217 + GFAP | | | 102 | +0.289 | 0.004 |
| ΔMMSE/year | + baseline MMSE | | | 102 | +0.169 | 0.095 |
| ΔMMSE/year | + baseline MMSE + age | | | 102 | +0.148 | 0.145 |
| ΔCDR-SB/year | Unadjusted | | | 102 | −0.473 | <0.001 |
| ΔCDR-SB/year | Adjusted for p-tau217 + GFAP | | | 102 | −0.389 | <0.001 |
| ΔCDR-SB/year | + baseline MMSE | | | 102 | −0.250 | 0.013 |
| ΔCDR-SB/year | + baseline MMSE + age | | | 102 | −0.241 | 0.017 |
| **Panel B. Within ADD stratum (continuous) (iMG NCKAP1)** | | | | | | |
| **Outcome** | **Adjustment model** | | | **n** | **Partial Spearman *r*** | ***p* value** |
| ΔMMSE/year | Adjusted for p-tau217 + GFAP | | | 76 | +0.167 | 0.155 |
| ΔMMSE/year | + baseline MMSE | | | 76 | +0.083 | 0.486 |
| ΔCDR-SB/year | Adjusted for p-tau217 + GFAP | | | 76 | −0.292 | 0.012 |
| ΔCDR-SB/year | + baseline MMSE | | | 76 | −0.222 | 0.059 |
| **Panel C. Multivariable linear regression (standardized), outcome = ΔMMSE/year** | | | | | | |
| **Outcome** | **Adjustment model** | | | **n** | **NCKAP1 β (standardized)** | ***p* value** |
| ΔMMSE/year | p-tau217 + GFAP | | | 102 | +0.274 | 0.004 |
| ΔMMSE/year | + baseline MMSE | | | 102 | +0.207 | 0.040 |
| ΔMMSE/year | + baseline MMSE + age | | | 102 | +0.187 | 0.057 |
| **Panel D. Stage-dependence of baseline predictors for functional progression (ΔCDR-SB/year; n ≈ 100)** | | | | | | |
| **Baseline predictor** | **Unadjusted** | **+ p-tau217 + GFAP** | **+ baseline MMSE** | | **+ baseline MMSE**  **+ age** | **+ baseline MMSE**  **+ age+ p-tau217 + GFAP** |
| iMG Aβ42 phago index | −0.334 (<0.001) | −0.199 (0.047) | −0.063 (0.534) | | −0.090 (0.380) | −0.090 (0.380) |
| iMG bead phago index | −0.276 (0.005) | −0.144 (0.154) | +0.035 (0.730) | | +0.017 (0.870) | +0.017 (0.870) |
| iMG E. coli phago index | −0.306 (0.002) | −0.224 (0.025) | +0.004 (0.971) | | +0.000 (0.999) | +0.000 (0.999) |
| iMG NCKAP1 | −0.473 (<0.001) | −0.389 (<0.001) | −0.250 (0.013) | | −0.241 (0.017) | −0.241 (0.017) |
| Plasma miR-214-3p | +0.525 (<0.001) | +0.449 (<0.001) | +0.335 (<0.001) | | +0.332 (<0.001) | +0.332 (<0.001) |
| Plasma p-tau217 | +0.277 (0.006) | — | +0.178 (0.081) | | +0.179 (0.081) | +0.190 (0.065) |
| Plasma GFAP | +0.206 (0.042) | — | +0.027 (0.793) | | +0.048 (0.644) | −0.081 (0.433) |
| Plasma NfL | +0.176 (0.083) | +0.002 (0.984) | +0.035 (0.732) | | +0.091 (0.377) | +0.006 (0.957) |

Panels A–C report partial Spearman correlations (A, B) and standardized multivariable linear-regression coefficients (C) between baseline iMG NCKAP1 expression and annualized clinical change. Positive coefficients for ΔMMSE/year and negative coefficients for ΔCDR-SB/year indicate slower clinical decline.

Panel D reports partial Spearman correlations between baseline biomarkers and ΔCDR-SB/year under progressive adjustment for baseline cognition, age, plasma p-tau217, and GFAP. For models including both plasma p-tau217 and GFAP as covariates, the predictor of interest was omitted from its own covariate set (e.g., p-tau217 models were adjusted for baseline cognition, age, and GFAP only). Corresponding analyses for ΔMMSE/year are presented in Figure 4 and the main text.

*Abbreviations*: ADD, amyloid-positive Alzheimer's disease dementia; GFAP, glial fibrillary acidic protein; iMG, induced microglia-like cell; miR-214-3p, microRNA-214-3p; MMSE, Mini-Mental State Examination; NCKAP1, NCK-associated protein 1; p-tau217, phosphorylated tau 217.

**Table S3. Clinical and biomarker characteristics according to microglial phagocytic dysfunction status in amyloid-positive AD dementia.**

|  | **NCKAP1-defined exhaustion (low NCKAP1)** | | | **miR-214-defined exhaustion (high miR-214)** | | |
| --- | --- | --- | --- | --- | --- | --- |
| **Characteristic** | **Non-exhausted ADD (high NCKAP1, n=38)** | **Exhausted ADD (low NCKAP1, n=38)** | ***p*** | **Non-exhausted ADD (low miR-214, n=38)** | **Exhausted ADD (high miR-214, n=38)** | ***p*** |
| **Demographics** | | | | | | |
| Age, years | 75 [68–79] | 74 [66–79] | 0.516 | 77 [70–80] | 72 [67–79] | 0.259 |
| Female sex | 16/38 (42.1%) | 24/38 (63.2%) | 0.107 | 17/38 (44.7%) | 23/38 (60.5%) | 0.251 |
| APOE ε4 carrier | 17/38 (44.7%) | 15/38 (39.5%) | 0.817 | 16/38 (42.1%) | 16/38 (42.1%) | 1.000 |
| **Baseline clinical status** | | | | | | |
| MMSE | 18.5 [15.5–21.0] | 14.0 [11.0–18.0] | 0.005 | 18.0 [14.0–21.0] | 15.5 [12.0–18.0] | 0.093 |
| CDR global score | 1.00 [0.62–1.00] | 1.00 [1.00–2.00] | 0.012 | 1.00 [0.62–1.00] | 1.00 [1.00–2.00] | 0.041 |
| CDR-SB | 5.5 [4.1–8.8] | 6.8 [4.6–10.8] | 0.116 | 5.5 [4.5–8.8] | 7.0 [5.0–10.8] | 0.093 |
| **Plasma biomarkers (same blood draw as iMGs)** | | | | | | |
| p-tau217, pg/mL | 1.04 [0.79–1.67] | 1.27 [1.05–1.61] | 0.203 | 1.24 [0.93–1.66] | 1.12 [0.86–1.55] | 0.380 |
| GFAP, pg/mL | 179 [141–247] | 216 [155–260] | 0.125 | 192 [150–258] | 197 [141–250] | 0.971 |
| NfL, pg/mL | 32.2 [19.8–42.0] | 34.3 [25.6–55.1] | 0.251 | 34.3 [23.6–57.1] | 32.0 [24.5–43.2] | 0.536 |
| Aβ42/40 ratio | 0.043 [0.037–0.049] | 0.045 [0.040–0.049] | 0.439 | 0.043 [0.039–0.050] | 0.043 [0.040–0.049] | 0.888 |
| p-tau217/Aβ42 ratio | 0.229 [0.159–0.386] | 0.287 [0.207–0.336] | 0.342 | 0.252 [0.178–0.334] | 0.274 [0.192–0.347] | 0.600 |
| **iMG functional and molecular markers (same blood draw)** | | | | | | |
| Aβ42 phagocytosis | 0.98 [0.81–1.40] | 0.94 [0.73–1.25] | 0.421 | 0.97 [0.73–1.31] | 0.96 [0.78–1.16] | 0.876 |
| Bead phagocytosis | 51.8 [37.3–70.0] | 46.5 [34.0–54.8] | 0.135 | 52.1 [36.6–62.7] | 48.7 [33.3–62.1] | 0.442 |
| E. coli phagocytosis | 57.8 [49.7–68.3] | 48.7 [35.4–58.3] | 0.019 | 55.0 [41.1–66.5] | 55.6 [40.3–64.9] | 0.946 |
| NCKAP1 (RQ) | 0.97 [0.79–1.22] | 0.40 [0.30–0.51] | <0.001 | 0.67 [0.41–0.98] | 0.66 [0.39–0.85] | 0.815 |
| Rubicon (RQ) | 0.97 [0.38–1.98] | 0.74 [0.36–4.23] | 1.000 | 0.73 [0.37–3.94] | 0.95 [0.32–4.13] | 0.846 |
| miR-214-3p (RQ) | 3.74 [2.08–5.35] | 3.63 [2.14–5.11] | 0.954 | 2.08 [1.50–2.72] | 5.27 [4.50–6.96] | <0.001 |
| **Longitudinal clinical progression** | | | | | | |
| ΔMMSE/yr | -1.59 [-2.74–-0.70] | -2.15 [-3.04–-1.03] | 0.287 | -1.34 [-2.21–-0.59] | -2.76 [-4.08–-1.36] | <0.001 |
| ΔCDR-SB/yr | 1.48 [0.69–3.02] | 1.95 [1.45–3.62] | 0.055 | 1.59 [0.74–2.40] | 2.06 [1.10–5.21] | 0.048 |
| Follow-up, years | 3.21 [2.50–4.17] | 2.91 [1.94–4.07] | 0.467 | 3.36 [2.66–4.19] | 2.71 [1.72–3.91] | 0.062 |
| rpAD (ΔMMSE/yr <−4 and ΔCDR-SB/yr ≥+2.5) ‡ | 5/38 (13.2%) | 4/38 (10.5%) | 1.000 | 0/38 (0.0%) | 9/38 (23.7%) | 0.002 |
| ΔMMSE/yr, adjusted † | ref | β=-0.43 | 0.397 | ref | β=-1.02 | 0.046 |

Participants with amyloid-positive AD dementia (ADD, n=76) were stratified at the median of iMG NCKAP1 expression or plasma miR-214-3p. Data are median [IQR] or n/N (%). Group comparisons used the Mann–Whitney U test (continuous) and Fisher exact test (categorical).

† Annualized MMSE change (ΔMMSE/year) was analyzed by linear regression. For NCKAP1, the association was attenuated after adjustment for baseline MMSE and age (β=−0.43, *p*=0.397). For plasma miR-214-3p, the association remained significant after adjustment for baseline MMSE, age, plasma p-tau217, and follow-up duration (β=−1.02, *p*=0.046).

‡ Fisher exact test. No participant in the low-miR-214-3p subgroup met rpAD criteria, precluding adjusted logistic regression because of complete separation.

*Abbreviations*: APOE ε4, apolipoprotein E ε4; Aβ42, amyloid-β 42; CDR, Clinical Dementia Rating; CDR-SB, CDR Sum of Boxes; GFAP, glial fibrillary acidic protein; iMG, induced microglia-like cell; IQR, interquartile range; miR-214-3p, microRNA-214-3p; MMSE, Mini-Mental State Examination; NCKAP1, NCK-associated protein 1; NfL, neurofilament light chain; p-tau217, phosphorylated tau 217; RQ, relative quantification.

**Table S4. Incremental prognostic performance of iMG measures beyond the clinical/biomarker base model.**

| **Outcome** | **Added iMG marker** | **n** | **C-index (base)** | **C-index (+iMG)** | **ΔC-index (95% CI)** | **LRT *p*** | **ΔAdj R²** |
| --- | --- | --- | --- | --- | --- | --- | --- |
| Clinical stage (CU<MCI<ADD) | Phagocytic composite | 114 | 0.835 | 0.912 | +0.078 (+0.031, +0.146) | <0.001 | +0.137 |
| Clinical stage (CU<MCI<ADD) | NCKAP1 | 114 | 0.834 | 0.853 | +0.019 (−0.002, +0.058) | 0.006 | +0.023 |
| ΔMMSE/year —full cohort | miR-214-3p | 102 | 0.645 | 0.712 | +0.066 (+0.013, +0.113) | <0.001 | +0.109 |
| ΔMMSE/year —full cohort | NCKAP1 | 102 | 0.645 | 0.678 | +0.033 (−0.003, +0.074) | 0.002 | +0.063 |
| ΔMMSE/year —ADD | miR-214-3p | 76 | 0.592 | 0.668 | +0.076 (+0.010, +0.138) | <0.001 | +0.116 |
| ΔMMSE/year —ADD | NCKAP1 | 76 | 0.592 | 0.640 | +0.048 (−0.008, +0.100) | 0.002 | +0.087 |
| ΔCDR-SB/year —full cohort | miR-214-3p | 102 | 0.677 | 0.682 | +0.005 (−0.008, +0.040) | 0.057 | +0.011 |
| ΔCDR-SB/year —ADD | miR-214-3p | 76 | 0.611 | 0.615 | +0.005 (−0.021, +0.059) | 0.015 | +0.040 |

Base models included age, log-transformed plasma p-tau217, and log-transformed plasma GFAP. Models for ΔMMSE/year additionally included baseline MMSE, and models for ΔCDR-SB/year additionally included baseline CDR-SB. Individual iMG measures (log miR-214-3p, NCKAP1 expression, or the phagocytic composite) were added separately to evaluate incremental predictive value.

The phagocytic composite was defined as the standardized mean of z-scored Aβ42, bead, and E. coli phagocytic indices. Discrimination was assessed using Harrell's concordance index (C-index) with optimism correction by bootstrap resampling (1,000 iterations). ΔC-index values and 95% confidence intervals were derived from the bootstrap distribution; CIs are method- and seed-dependent. Likelihood-ratio test (LRT) *p* values (one-sided) and changes in adjusted R² were obtained from nested regression models. Analyses are internal and exploratory.

*Abbreviations*: ADD, amyloid-positive Alzheimer's disease dementia; Aβ42, amyloid-β 42; CDR-SB, CDR Sum of Boxes; CI, confidence interval; CU, cognitively unimpaired; GFAP, glial fibrillary acidic protein; iMG, induced microglia-like cell; MCI, amyloid-positive mild cognitive impairment; miR-214-3p, microRNA-214-3p; MMSE, Mini-Mental State Examination; NCKAP1, NCK-associated protein 1; p-tau217, phosphorylated tau 217.

**Table S5. Baseline iMG and plasma biomarker profiles of amyloid-positive MCI participants who converted to dementia versus non-converters.**

| **Characteristic** | **Converters (n=5)** | **Non-converters (n=13)** | ***p* value** |
| --- | --- | --- | --- |
| Age (years) | 77 [76–85] | 71 [66–78] | 0.299 |
| Baseline MMSE | 20.0 [20.0–24.0] | 26.0 [25.0–27.0] | 0.037 |
| Baseline CDR-SB | 2.0 [1.5–2.0] | 1.0 [0.5–1.5] | 0.116 |
| iMG NCKAP1 (relative) | 0.89 [0.64–0.96] | 0.96 [0.79–1.30] | 0.300 |
| iMG Rubicon (relative) | 1.98 [0.96–13.27] | 7.05 [0.82–10.64] | 0.849 |
| iMG Aβ42 phagocytic index | 1.34 [1.16–1.49] | 1.74 [1.33–2.40] | 0.443 |
| iMG bead phagocytic index | 68.9 [52.2–85.6] | 72.3 [64.6–82.5] | 0.849 |
| iMG E. coli phagocytic index | 68.1 [66.4–82.7] | 68.2 [63.6–81.7] | 0.490 |
| Plasma miR-214-3p (relative) | 1.92 [1.23–2.86] | 1.47 [1.27–1.66] | 0.503 |
| Plasma p-tau217 (pg/mL) | 1.33 [1.31–1.97] | 0.88 [0.76–1.05] | 0.143 |
| Plasma GFAP (pg/mL) | 153.3 [105.4–336.5] | 136.7 [99.6–166.6] | 0.503 |
| Plasma NfL (pg/mL) | 37.6 [37.6–58.6] | 20.3 [16.2–30.5] | 0.117 |

Data are presented as median [IQR]. *P* values were calculated using two-sided Mann–Whitney U tests.

Converters (n=5) were amyloid-positive MCI participants whose baseline global Clinical Dementia Rating (CDR) score of 0.5 progressed to ≥1 during follow-up. The remaining amyloid-positive MCI participants (n=13) were classified as non-converters.

Significant differences were observed only for baseline MMSE. No baseline iMG functional measure or plasma biomarker reached statistical significance.

*Abbreviations*: Aβ42, amyloid-β 42; CDR-SB, CDR Sum of Boxes; GFAP, glial fibrillary acidic protein; iMG, induced microglia-like cell; MCI, amyloid-positive mild cognitive impairment; miR-214-3p, microRNA-214-3p; MMSE, Mini-Mental State Examination; NCKAP1, NCK-associated protein 1; NfL, neurofilament light chain; p-tau217, phosphorylated tau 217.

**Table S6. Longitudinal changes in iMG and plasma markers and their associations with cognitive decline.**

| **Marker** | **n (subjects)** | **n (obs.)** | **MLM slope (SD/year)** | **95% CI** | **MLM *p*** | **Paired Wilcoxon p** | **Δ/year vs ΔMMSE/year (Spearman *r*, *p*)** |
| --- | --- | --- | --- | --- | --- | --- | --- |
| iMG NCKAP1 | 38 | 76 | -0.157 | [-0.234, -0.079] | <0.001 *** | <0.001 | +0.47 (0.003) |
| Plasma miR-214-3p (log) | 38 | 76 | +0.115 | [+0.055, +0.176] | <0.001 *** | <0.001 | -0.67 (<0.001) |
| iMG bead phagocytosis | 38 | 76 | -0.106 | [-0.192, -0.020] | 0.015 * | 0.007 | +0.34 (0.036) |
| Plasma p-tau217 | 38 | 76 | +0.045 | [-0.021, +0.110] | 0.182 n.s. | <0.001 | -0.00 (0.994) |
| Plasma GFAP | 38 | 76 | +0.089 | [+0.011, +0.168] | 0.026 * | 0.011 | -0.05 (0.782) |
| Plasma NfL | 38 | 76 | +0.007 | [-0.031, +0.044] | 0.731 n.s. | 0.204 | +0.07 (0.663) |

Longitudinal biomarker cohort (amyloid-positive primary continuum; 38 participants with paired baseline and follow-up samples; 76 observations). Linear mixed-effects models (MLM) fitted as standardized marker ~ time (years from baseline) + random intercept per participant; slopes are in standard-deviation units per year (negative = decline). Only the right-skewed marker miR-214-3p was log-transformed before standardization. Paired Wilcoxon p compares baseline vs follow-up. The final column reports Spearman correlation between annualized biomarker change (Δ/year) and annualized MMSE change. Microglial markers (NCKAP1 decline, miR-214-3p rise, bead phagocytosis decline) were dynamically coupled to cognitive decline, whereas plasma p-tau217/GFAP/NfL were not. Plasma p-tau217 increased baseline-to-follow-up (paired Wilcoxon *p*<0.001) but its per-year slope was not significant in the mixed-effects model (*p*=0.18) and was uncoupled from cognitive tempo. **p*<0.05, ***p*<0.01, ****p*<0.001.

*Abbreviations*: CI, confidence interval; GFAP, glial fibrillary acidic protein; iMG, induced microglia-like cell; MLM, linear mixed-effects model; miR-214-3p, microRNA-214-3p; MMSE, Mini-Mental State Examination; NCKAP1, NCK-associated protein 1; NfL, neurofilament light chain; p-tau217, phosphorylated tau 217; SD, standard deviation.

**Table S7. Age associations and age-adjusted analyses of microglial markers.**

| **Variable / comparison** | **n** | **Statistic** | ***p*-value** |
| --- | --- | --- | --- |
| **A. Univariate association with age (Spearman's ρ)** | | | |
| iMG Aβ42 phagocytosis | 112 | ρ = -0.350 | <0.001 *** |
| iMG bead phagocytosis | 112 | ρ = -0.336 | <0.001 *** |
| iMG E. coli phagocytosis | 112 | ρ = -0.216 | 0.022 * |
| iMG NCKAP1 | 112 | ρ = -0.118 | 0.217 n.s. |
| Plasma miR-214-3p | 112 | ρ = +0.099 | 0.298 n.s. |
| Plasma NfL | 112 | ρ = +0.534 | <0.001 *** |
| Plasma GFAP | 112 | ρ = +0.362 | <0.001 *** |
| Plasma p-tau217 | 112 | ρ = +0.241 | 0.011 * |
| **B. Between-stage difference after age residualization (Kruskal–Wallis on age-regressed residuals; HC / A+MCI / A+ADD)** | | | |
| iMG Aβ42 phagocytosis | 18/18/76 | H = 26.8 | <0.001 ******* |
| iMG bead phagocytosis | 18/18/76 | H = 37.4 | <0.001 ******* |
| iMG E. coli phagocytosis | 18/18/76 | H = 35.7 | <0.001 ******* |
| iMG NCKAP1 | 18/18/76 | H = 24.5 | <0.001 ******* |
| **C. Partial correlation adjusting for age (residual method)** | | | |
| Plasma GFAP × iMG Aβ42 phagocytosis | 112 | partial *r* = -0.433 (ρ = -0.505) | <0.001 *** |
| Plasma GFAP × iMG bead phagocytosis | 112 | partial *r* = -0.437 (ρ = -0.505) | <0.001 *** |
| Plasma p-tau217 × iMG Aβ42 phagocytosis | 112 | partial *r* = -0.311 (ρ = -0.367) | <0.001 *** |
| iMG NCKAP1 × Plasma miR-214-3p | 112 | partial *r* = -0.345 (ρ = -0.352) | <0.001 *** |

All analyses were performed in the primary cohort (HC + amyloid-positive MCI + amyloid-positive ADD; n=112); the amyloid-positive CU reference group (n=2) was excluded.(A) Spearman rank correlation between age and each marker. (B) Each marker was regressed on age and the residuals compared across stages by Kruskal–Wallis, testing whether stage effects persist independently of age. (C) Partial correlations between plasma and iMG markers after regressing out age from both variables (Pearson partial r shown, with Spearman partial ρ in parentheses).

Significance: * *p*<0.05, ** *p*<0.01, *** *p*<0.001. Age is associated with iMG phagocytic capacity and plasma NfL/GFAP, but not with iMG NCKAP1 or plasma miR-214-3p, and stage-dependent differences in phagocytosis and NCKAP1 remain highly significant after age adjustment — indicating that the NCKAP1/miR-214 axis and disease-stage effects are age-independent.
